# Polygonal surface processing and mesh generation tools for the numerical simulation of the cardiac function

**DOI:** 10.1002/cnm.3435

**Published:** 2021-01-28

**Authors:** Marco Fedele, Alfio Quarteroni

**Affiliations:** ^1^ MOX, Department of Mathematics Politecnico di Milano Milan Italy; ^2^ Institute of Mathematics École Polytechnique Fédérale de Lausanne Lausanne Switzerland

**Keywords:** cardiac mesh generation, heart modeling, patient‐specific modeling, polygonal surface processing

## Abstract

In order to simulate the cardiac function for a patient‐specific geometry, the generation of the computational mesh is crucially important. In practice, the input is typically a set of unprocessed polygonal surfaces coming either from a template geometry or from medical images. These surfaces need ad‐hoc processing to be suitable for a volumetric mesh generation. In this work we propose a set of new algorithms and tools aiming to facilitate the mesh generation process. In particular, we focus on different aspects of a cardiac mesh generation pipeline: (1) specific polygonal surface processing for cardiac geometries, like connection of different heart chambers or segmentation outputs; (2) generation of accurate boundary tags; (3) definition of mesh‐size functions dependent on relevant geometric quantities; (4) processing and connecting together several volumetric meshes. The new algorithms—implemented in the open‐source software *vmtk*—can be combined with each other allowing the creation of personalized pipelines, that can be optimized for each cardiac geometry or for each aspect of the cardiac function to be modeled. Thanks to these features, the proposed tools can significantly speed‐up the mesh generation process for a large range of cardiac applications, from single‐chamber single‐physics simulations to multi‐chambers multi‐physics simulations. We detail all the proposed algorithms motivating them in the cardiac context and we highlight their flexibility by showing different examples of cardiac mesh generation pipelines.

## Graphical abstract

1. We propose a set of algorithms and tools aiming to facilitate and speed‐up the mesh generation process for cardiac geometries.
2. The main novelties regard the polygonal surface processing, the boundary tags definition, the mesh‐size definition, and the volumetric mesh processing.
3. The new algorithms can be combined with each other to create personalized application‐specific pipelines.
4. We demonstrate the robustness and the flexibility of the proposed tools through various examples on different kinds of cardiac geometries.

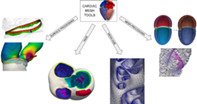

## 1 INTRODUCTION

The mathematical and numerical modeling of the cardiac function is a very challenging research topic.^1^ Advances in this field can help to better understand the heart physiology^2^, ^3^, ^4^, ^5^, ^6^, ^7^ and are promoting a new approach to the study of cardiac pathologies and their treatments.^8^, ^9^, ^10^, ^11^, ^12^, ^13^, ^14^

In terms of modeling, the multi‐physics aspects of the cardiac function can be grouped into three main processes: the *electrophysiology* that takes into account the electric signal propagation and the biochemical processing occurring at cells level; the active and passive mechanics that, driven by the electric signal and by the pressure evolution inside the cardiac chambers, models the movement and the internal stresses of the cardiac muscle; and the fluid‐dynamics that, together with the motion of the four cardiac valves, regulates the blood flow circulation in the entire human body.

**FIGURE 1:**
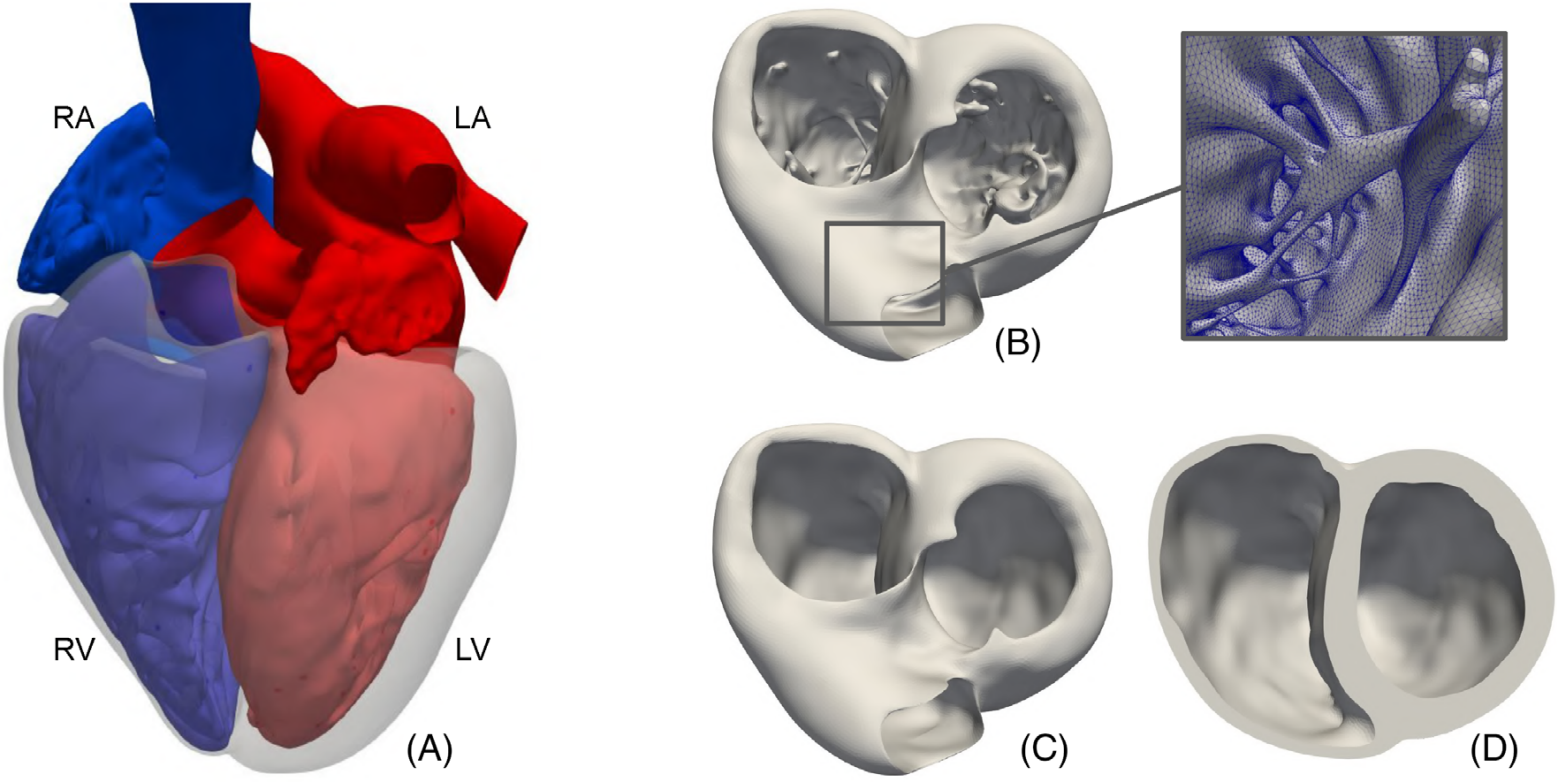
On the left (A), an example of heart geometry, elaborated from the Zygote Solid Heart model^15^: the Left Atrium (LA) and the Left Ventricle (LV) are filled up with oxygenated blood (in red), while the right atrium (RA) and the right ventricle (RV) are full of non‐oxygenated blood (in blue); the ventricular myocardial muscle is shown in transparency, while the atrial muscle—being very thin—is not depicted. On the right (B‐D), the same ventricular geometry depicted with a decreasing level of details; for the most detailed one (B) an inside zoom of the papillary muscles and trabeculae carneae is also shown

From the geometric point of view, the heart is characterized by a very complex anatomy, as illustrated in Figure 1A. Both the ventricular and the atrial muscles are in continuity from the left to the right side of the heart, while they are connected to each other only by the fibrotic tissue of the annuli, which also acts as electrical insulators.^16^ On the contrary, from the hemodynamic point of view, the internal cavities are connected only along the atrioventricular direction, while a clear separation between the right and the left cavities is necessary to divide the oxygenated and the non‐oxygenated blood.^16^ Thus, from the geometric point of view, a complete electro‐mechano‐fluid model of the cardiac function must take into account both the atrio‐ventricular muscle separation and the right–left hemodynamic division. The complexity is furtherly increased by the presence on the endocardium of the papillary muscles and the trabeculae carneae, which make the inner part of the heart a rough and irregular surface, as shown for the ventricles in Figure 1B. Depending on the focus of the study, this geometric complexity can be addressed with a different level of detail, see for example, Figure 1B–D.

Starting from these general considerations, in the literature we can find a variety of computational studies focused on completely different aspects of the cardiac function: standalone electrophysiology simulations^17^, ^18^, ^19^ often focused on particular pathologies and clinical applications^13^, ^20^; electromechanics studies^2^, ^7^, ^21^, ^22^; fluid‐dynamics simulations involving the cardiac valve modeling^23^, ^24^, ^25^, ^26^, ^27^, ^28^; and, more seldom, full electro‐mechano‐fluid simulations of the heart, albeit with some simplifications.^29^, ^30^

A computational study of the cardiac function, especially in the case of patient‐specific geometries reconstructed from medical images, generally starts from the processing of polygonal surfaces and the generation of a computational mesh. The difficulty of this first pre‐processing step increases with the number of cardiac chambers we aim to address and the level of geometric detail desired. On the one hand, the mesh generation of a smooth left‐ventricle cut at the base (see Figure 1D) can be considered a relatively simple process. On the other hand, dealing with detailed cardiac geometries (see Figure 1B) or with the constraint of creating conforming meshes between the different chambers of the heart can be a very challenging time‐ and manpower‐consuming operation.

Mesh generation is a field characterized by very different techniques. A detailed review of this topic goes beyond the aim of this paper and can be found, for instance, in References 31, 32, 33, 34, 35 Concerning the cardiac field, various application‐specific pipelines have been proposed in literature.^36^, ^37^, ^38^, ^39^, ^40^, ^41^, ^42^, ^43^, ^44^, ^45^, ^46^

In this context, grid‐based techniques^47^ invariably start from an initial regular grid—for example, the medical image voxel grid—and adapt the mesh till matching the desired geometry—for example, a multi‐label image segmentation. Exploiting such kind of techniques, Prassl et al^38^ proposed a pipeline to build detailed biventricular geometries for electrophysiology models. Similarly, Strocchi et al^45^ generated a cohort of four‐chambers meshes for electro‐mechanical models from high‐resolution CT‐scans, exploiting the integration in the pipeline of an automatic multi‐label segmentation method.^48^ As a drawback, these methods all rely on a specific kind of image data or segmentation technique.^38^ Thus, they are not applicable, for instance, in the case where each chamber is reconstructed from a different image.

Another technique consists in the use of an application‐specific template mesh, that is deformed into each patient‐specific geometry.^39^, ^40^, ^46^, ^49^ The template can be an idealized geometry^39^ or an atlas built from a large dataset of medical images.^50^, ^51^, ^52^, ^53^ In this context, Lamata et al,^39^ using an idealized biventricular template, proposed a pipeline to generate smooth biventricular hexahedral meshes for mechanics simulations; This et al^46^ exploit a smooth template geometry of the left‐heart endocardium to perform patient‐specific hemodynamics simulations; Hoogendoorn et al^49^ adopt an atlas‐based method to perform patient‐specific electrophysiology simulations in smooth biventricular geometries; Zhang et al^40^ proposed an atlas‐based method to build cubic Hermite finite element meshes of a full‐heart smooth geometry. Recently, template‐based techniques have also been proposed in the context of isogeometric analysis.^54^, ^55^ However, all these techniques strongly rely on the features of the template geometry and are strictly related to the application they are designed for. Additionally, they may have difficulty capturing detailed cardiac geometries, due to the significant anatomical variations of the heart.^56^, ^57^, ^58^

In the context of patient‐specific cardiovascular applications, surface‐based techniques such as advancing‐fronts^59^, ^60^, ^61^ and Delaunay‐based methods^62^ still remain very popular. These methods discretize the domain starting from the external polygonal surface and propagate the mesh‐size of the polygonal elements towards the inside of the volume. The main advantage is a full control over the meshing process through, for instance, coarsening or refinement of specific parts of the surface. Moreover, since they can start from the output of any medical image segmentation algorithm, they can accurately reproduce detailed patient‐specific geometries of both healthy or pathological hearts. This is of fundamental importance since, among all the parameters considered for a cardiovascular simulation, recent studies demonstrate how the geometry has a significant impact on the outputs.^63^, ^64^, ^65^ As a drawback, these methods require triangulated surfaces of sufficient good quality, whereas thin or intersecting surfaces, typical of cardiac segmentation outputs, can be problematic. Consequently, flexible and easy‐to‐use algorithms to process polygonal surfaces are required to make surface‐based techniques more powerful for cardiac mesh generation.

**FIGURE 2:**
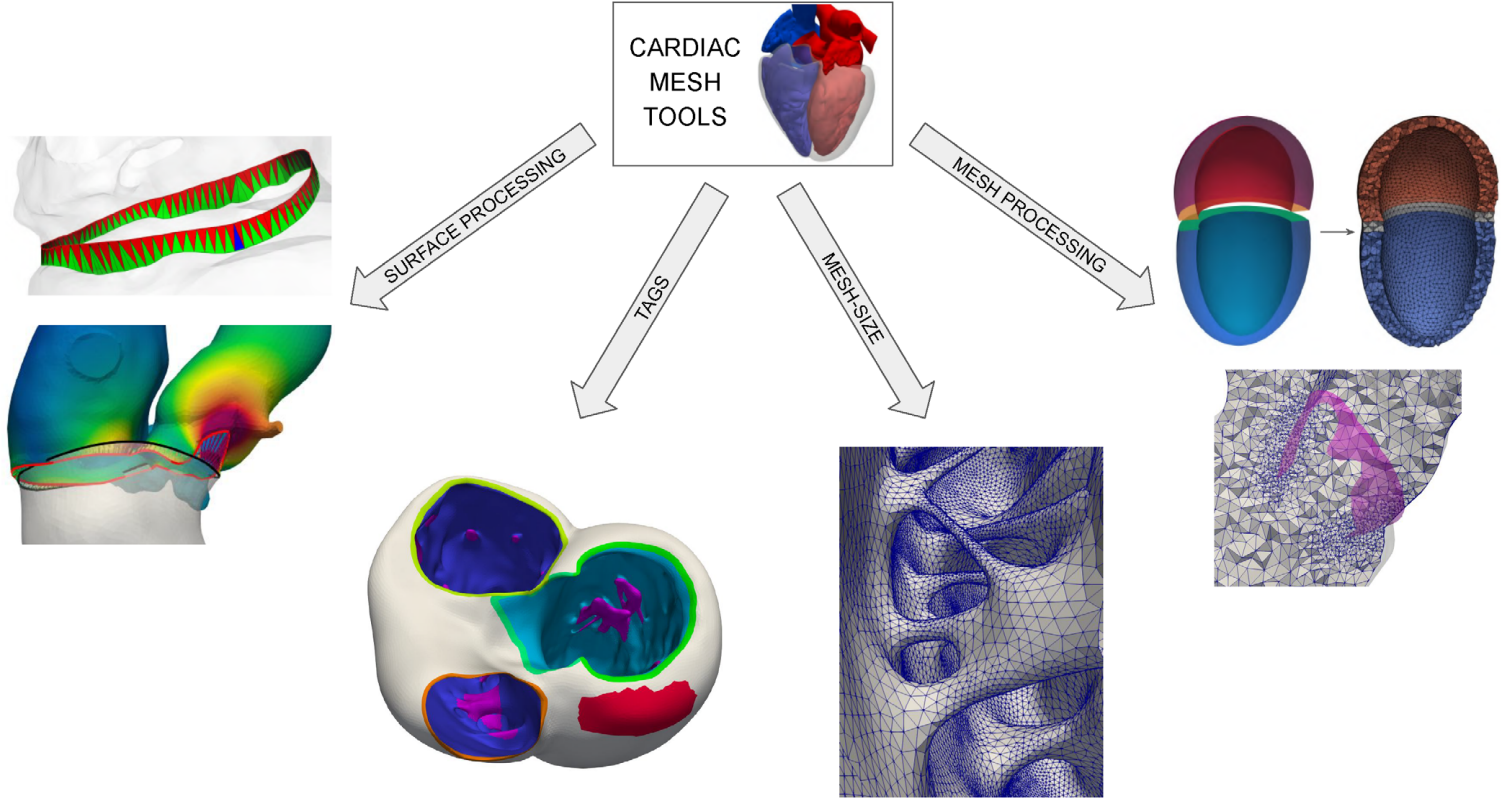
A sketch of the cardiac mesh tools covered in this paper, highlighting the four groups into which they are divided

Summarizing, a unique mesh generation pipeline for all the cardiac applications cannot hardly proposed. For this reason, in this paper, in the context of surface‐based techniques, we propose various independent algorithms and tools that can be combined with each other in a flexible way, creating application‐dependent pipelines. In particular, we start from stable algorithms and tools originally designed for vascular surface processing and mesh generation and implemented in the vascular modeling toolkit (vmtk)^*^ library^66^ and we extend them with new specific algorithms developed for cardiac mesh generation. As sketched in Figure 2, the proposed algorithms will address four different tasks: polygonal surface processing; boundary tags definition; mesh‐size definition; volumetric mesh processing. For each of these tasks, we propose new algorithms to facilitate and automatize common steps in a cardiac mesh generation pipeline, overcoming the typical problems related to surface‐based techniques and cardiac mesh generation. The proposed algorithms—publicly available in a fork of vmtk^†^—are released in a unique framework that allows tremendous flexibility in the creation of mesh‐generation pipelines designed for the needs of the specific cardiac model. Thus, the proposed framework can be used to create single‐ or multi‐chamber geometries with different levels of details, depending on the kind of simulation to be performed. This feature can be considered the primary contribution of our paper. Indeed, despite the existence of numerous open‐source or commercial softwares in this area (e.g., *Gmsh*,^67^ *Netgen*,^68^ *Cubit*,^69^ *MeshLab*,^70^ *Meshmixer*,^71^ *Meshtool*,^44^ *Blender* ^72^), having all the tools required to build a cardiac computational mesh in a single pipeline can significantly speed‐up this time‐ and manpower‐consuming operation.

Moreover, the possibility of creating fully‐automatic pipelines for a specific application can also be exploited for clinical studies on huge datasets or to create virtual cohorts of heart models.^73^

The outline of the paper is as follows: in Section 2 we illustrate all the proposed algorithms, and for each of them we provide motivations through significant examples in the cardiac context; in Section 3 we combine the proposed algorithms in single pipelines to generate different kinds of cardiac meshes, and show some examples of already published numerical studies focused on different aspects of the cardiac function; a final discussion and the conclusions of our work are drawn in Section 4.

## 2 METHODS

In Table 1 we summarize all the new algorithms and tools proposed in this paper, underlining their main input and output. As sketched in Figure 2, we can group them into the following four macro‐areas, to each of which we will dedicate a specific section.

1. **Polygonal surface processing** (Section 2.1). Among the variety of algorithms related to this broad topic, we propose four algorithms designed for specific processing needs in the framework of cardiac applications. In particular, three of them—*surface‐connection* (Section 2.1.1), *boolean‐connection* (Section 2.1.2), and *harmonic‐connection* (Section 2.1.3)—concern different ways to join two separate polygonal surfaces. This operation is of paramount importance in cardiac applications, where the segmentation output can consist of multiple surfaces, for example, the endocardium and the epicardium of the ventricles or different cardiac chambers. Additionally, we also propose an algorithm—*surface‐thickening* (Section 2.1.4)—to post‐process a cardiac muscular mesh (e.g., an atrium or a ventricle) in order to locally modify its thickness.
2. **Boundary tags definition** (Section 2.2). While in most engineering applications the geometry can be defined as a CAD model^74^ and consequently the boundaries are identifiable as part of this model (e.g., by exploiting sharp edges), significant boundaries of cardiovascular geometries (e.g., valvular annuli) could also appear in smooth parts of the domain. In this context, we propose a flexible and precise tool—*surface‐tagger* (Section 2.2)—to automatically tag a surface by exploiting significant functions defined on it.
3. **Array processing and mesh‐size definition** (Section 2.3). While for vascular applications the mesh‐size is usually dependent on the local radius of the vessel,^66^, ^75^, ^76^ for complex cardiac geometries (see, for instance, Figure 1B) more than one geometric quantity needs to be considered (e.g., local curvature, muscle thickness). This topic is also linked to the definition of specific arrays on a surface and to their processing. In this context, we propose the *surface‐thickness* algorithm (Section 2.3.2) to compute the thickness of the cardiac muscle, and the harmonic‐extension algorithm (Section 2.3.1) to extend a field defined only on a part of the surface. Finally, we propose a tool—*surface‐mesh‐size* (Section 2.3.3)—to combine and manipulate different arrays in order to flexibly define the desired mesh‐size function on a surface.
4. **Volumetric mesh processing** (Section 2.4). In surface‐based techniques, after the generation of a surface mesh of the desired mesh‐size, the volumetric mesh generation is a straightforward operation based on well‐known algorithms.^60^, ^62^, ^68^, ^77^ However, for some cardiac applications, additional processing on the volumetric mesh could be necessary. For this purpose, we propose three algorithms: the *mesh‐connection* algorithm (Section 2.4.1) to connect two disconnected volumetric meshes, for example, two cardiac chambers at their valvular annuli; a local‐refinement algorithm—*mesh‐refinement* (Section 2.4.2)—based on the constrained Delaunay refinement method of *TetGen* ^62^; and the *tet‐hex* algorithm (Section 2.4.3) to include in our framework a well‐known strategy to convert tetrahedral meshes into hexahedral ones.

**TABLE 1:** List of all the proposed algorithms and tools with their main input and output

Name	Input	Output
*surface‐connection* (Section 2.1.1)	Two open surfaces separated by a gap	A continuous surface obtained by smoothly connecting two boundaries of the input surfaces
*Boolean‐connection* (Section 2.1.2)	Two intersecting surfaces	A continuous smooth surface made of regular triangles obtained as the union, the difference, or the intersection of the two inputs
*Harmonic‐connection* (Section 2.1.3)	An input surface and a reference surface	A continuous surface made of regular triangles obtained by deforming a boundary of the input surface into a boundary of the reference one, and extending the deformation through a harmonic map
*Surface‐thickening* (Section 2.1.4)	A surface with an array defined on it representing the thickness	A thickened surface in the regions where the input thickness is lower than a threshold
*Surface‐tagger* (Section 2.2)	A surface with geometric quantities defined on it as arrays	A tagged surface where the boundary tags are created by exploiting different algorithms based on either the manipulation of the input arrays or an interactive graphical interface
Harmonic‐extension (Section 2.3.1)	A surface with a scalar or vector field defined only on a subpart of it	A surface with the input field extended on the whole domain through a harmonic map
*Surface‐thickness* (Section 2.3.2)	A tagged surface with specific tags for the internal and the external walls	A surface with its thickness defined on it as an array
*Surface‐mesh‐size* (Section 2.3.3)	A surface with geometric quantities defined on it as arrays	A surface with an additional array representing the mesh‐size, computed by manipulating the input arrays
*mesh‐connection* (Section 2.4.1)	Two tagged volumetric meshes separated by a gap	A unique volumetric mesh created by generating a connecting volume between two selected regions of the two input meshes
*Mesh‐refinement* (Section 2.4.2)	A volumetric mesh with a geometric quantity or function defined on it	A volumetric mesh locally refined by exploiting the input array as a sizing function
*tet‐hex* (Section 2.4.3)	A tetrahedral volumetric mesh	A hexahedral volumetric mesh obtained by subdividing each tetrahedron intro four hexahedra

We conclude this introduction to the proposed algorithms by motivating the choice of *vmtk* as development environment and summarizing some notations that we will adopt in the following sections. *vmtk* is a library written in C++ and Python that can be used as a collection of Python scripts linkable to each other in a pipeline, meaning that the output of a script can be automatically used as the input of another script and so on. Moreover many algorithms and tools of *vmtk*—despite being originally thought for vascular modeling—can also be applied in the cardiac context. We mention in particular the remeshing algorithm, which is able to remesh a surface according to a mesh‐size function defined on it and preserving the geometry of the boundary tags. Moreover, the remeshing can be localized only on a subset of tags, allowing a significant speed‐up when dealing with complex geometries. In the following, the word “remeshing” always refers to the *vmtk* remeshing algorithm; we refer to Antiga et al^66^ for more details. Two other fundamental features of *vmtk* are the embedding of *TetGen* ^62^—for the volumetric mesh generation through an efficient version of the Delaunay algorithm^60^, ^77^—and its internal structure based on the *VTK* library.^78^ In particular, we use the latter to require user input through a 3D interactive graphical interface.

In the following algorithms, we deal with both 3D domains, 2D manifolds, and 1D curves, depending on the operation to be performed. We assign a specific Greek letter to each of these three cases, by denoting with Ω the 3D volumetric meshes, with ∑ the 2D polygonal surfaces, and with Γ the 1D curves. A closed 1D curve typically appears as a boundary of a polygonal surface. In this context, we often denote it as ring, since the boundary of the cardiovascular surfaces is usually ring‐shaped. If not otherwise specified, we suppose that Ω is made of tetrahedral elements, ∑ of triangles, and Γ of lines. We indicate a surface without boundary as closed surface, the opposite case as open surface. Finally, we denote as unsigned distance the positive scalar function representing the euclidean distance from a reference object and as signed distance the same distance with a positive or negative sign depending on whether the point is outside or inside the reference object, respectively. These distances are computed using the angle weighted pseudonormal algorithm implemented in *VTK*.^79^, ^80^

### 2.1 Polygonal surface processing

#### 2.1.1 Connection of disconnected surfaces: The *surface‐connection* algorithm

##### Motivations

The starting point of a patent‐specific mesh generation pipeline in the cardiovascular context can often be a set of disconnected surfaces separated by a gap. For instance, the generation of a mesh for the simulation of the fluid dynamics in the heart chambers can typically start from the disconnected internal surfaces of the right/left ventricle, the right/left atrium, and the various inlet/outlet vessels. Indeed, all these geometries can be reconstructed independently from different kinds of images and need to be merged in order to create a continuous triangulation. We will see a complete example of a pipeline to generate such kind of fluid‐dynamic mesh in Section 3, whereas here we detail the new algorithm proposed to connect two disconnected surfaces.

##### Input, output, parameters and options

The algorithm takes as input two non‐overlapping disconnected open surfaces—named ∑_1_ and ∑_2_. As output, we obtain a unique surface ∑ connecting the two input surfaces from a ring Γ_1_ ∈ ∑_1_ to a ring Γ_2_ ∈ ∑_2_ through a smooth continuous triangulation coherent with the shape of the two boundaries. The connecting triangulation is marked with a specific tag. No parameters are required since the algorithm is completely automatic; if ∑_1_ or ∑_2_ have more than a single boundary ring, a graphical interface allows the user to choose which one to connect.

##### The *surface‐connection* algorithm

**FIGURE 3:**
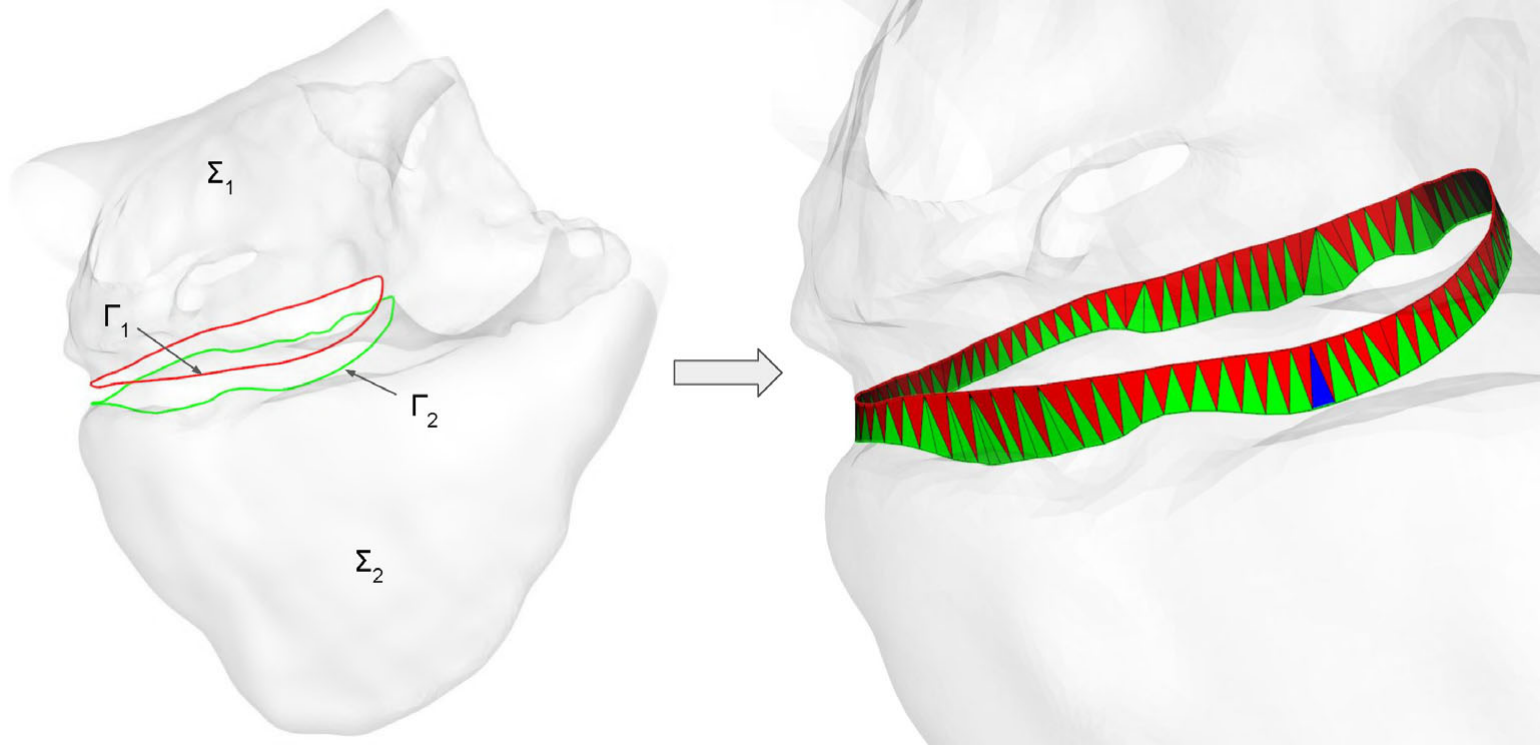
Connecting the right atrium (∑_1_) and the right ventricle (∑_2_) at the tricuspid valvular annulus (Γ_1_ and Γ_2_) using the *surface‐connection* algorithm. Each generated triangle lies on Γ_1_ (red triangles) or Γ_2_ (green‐triangles) depending on a minimum distance criterion, while a specific criterion is used for the last triangle that closes the triangulation (in blue)

Our algorithm starts from a random point on Γ_1_ and from its closest point on Γ_2_, then it iteratively adds a triangle on Γ_1_ or on Γ_2_ choosing always the triangle with the shortest connecting edge between the two rings. In Figure 3 we show an example where the right atrium ∑_1_ and the right ventricle ∑_2_ are connected at their boundaries Γ_1_ and Γ_2_, respectively, located at the tricuspid valve annulus. More in detail the algorithm proceeds as follows:

1. Extract from ∑_1_ and ∑_2_ all the boundaries, storing them into two sets of *N*_1_ and *N*_2_ rings, respectively;
2. select Γ_1_ and Γ_2_ from the two boundary sets, using a graphical interface if *N*_1_ > 1 or *N*_2_ > 1;
3. supposing that Γ_1_ and Γ_2_ are made of *n*_1_ and *n*_2_ points, respectively, select a random point $P_{1}^{0}$ on Γ_1_ and find the closest point $P_{2}^{0}$ on Γ_2_. These are the two starting points of the connecting triangulation, that at this stage is made of the set of points $P=\left\{P_{1}^{0},P_{2}^{0}\right\}$ and of an empty set of triangles T=∅;
4. find the two neighbor points of $P_{1}^{0}$ and select randomly one of them as $P_{1}^{*}$;
5. find the two neighbor points of $P_{2}^{0}$ and select as $P_{2}^{*}$ the closest point to $P_{1}^{*}$;
6. initializing $P_{1}=P_{1}^{0}$, $P_{2}=P_{2}^{0}$, and *i* = 2 as the counter of the points inserted in P, proceed as follows while *i* ≤ (*n*_1_ + *n*_2_):
  - compute the distances $d_{1}=|P_{1}^{*}-P_{2}|$ and $d_{2}=|P_{2}^{*}-P_{1}|$;
  - if *d*_1_ < *d*_2_, then set $P^{*}=P_{1}^{*}$ (red‐triangle case of Figure 3), else set $P^{*}=P_{2}^{*}$ (green‐triangle case of Figure 3);
  - insert *P* ^*^ into the set of points $P=P\cup \left\{P^{*}\right\}$ and increment *i* = *i* + 1;
  - create the triangle *T* made by the points (*P*_1_, *P*_2_, *P* ^*^) and insert it in the triangulation $T=T\cup \left\{T\right\}$;
  - if *d*_1_ < *d*_2_, then update *P*_1_ = *P* ^*^ and $P_{1}^{*}$ as the neighbor of *P*_1_ not yet inserted in P, otherwise do the same operation for *P*_2_ and $P_{2}^{*}$;
7. at the end of the cycle, P comprises (*n*_1_ + *n*_2_ + 1) points. Indeed, only one point between $P_{1}^{0}$ and $P_{2}^{0}$—that is, the two initial points—has been inserted twice. Thus, at this stage, $\left(P_{1}=P_{1}^{0}\right)⊻\left(P_{2}=P_{2}^{0}\right)$. In order to close the triangulation T, we have to insert the last triangle *T* ^*^—depicted in blue in Figure 3, right—exploiting the point between $P_{1}^{0}$ and $P_{2}^{0}$ that has not yet been inserted twice: if $P_{1}=P_{1}^{0}$, then *T* ^*^ is made of $\left(P_{1},P_{2},P_{2}^{0}\right)$, else *T* ^*^ is made of $\left(P_{1},P_{2},P_{1}^{0}\right)$.
8. assign a specific tag to the triangles of the just created connecting triangulation T;
9. merge T with the two input surfaces by removing duplicate points, creating a unique connected surface $\sum =\sum_{1}\cup T\cup \sum_{2}$.

##### Discussion

The proposed algorithm demonstrates its robustness when applied to complex cardiac geometries (see Section 3), since it works also when the distance between the rings Γ_1_ and Γ_2_ varies in a large range. Moreover, we remark that the two rings to be connected can be made of a very different number of points. Indeed, thanks to the minimum distance criterion, the algorithm will insert always the best possible triangle that maintains the smoothness of the connection. Clearly, the resulting triangulation T can be made of “slim” or “fat” triangles. However, using the capability of *vmtk* of remeshing only a single tag, the new triangulation can be easily remeshed with an arbitrary mesh‐size, recovering its regularity and, at the same time, maintaining both the shape of the triangulation T and the conformity with the two input surfaces ∑_1_ and ∑_2_.

#### 2.1.2 Connection of intersecting surfaces: The *boolean‐connection* algorithm

##### Motivations

**FIGURE 4:**
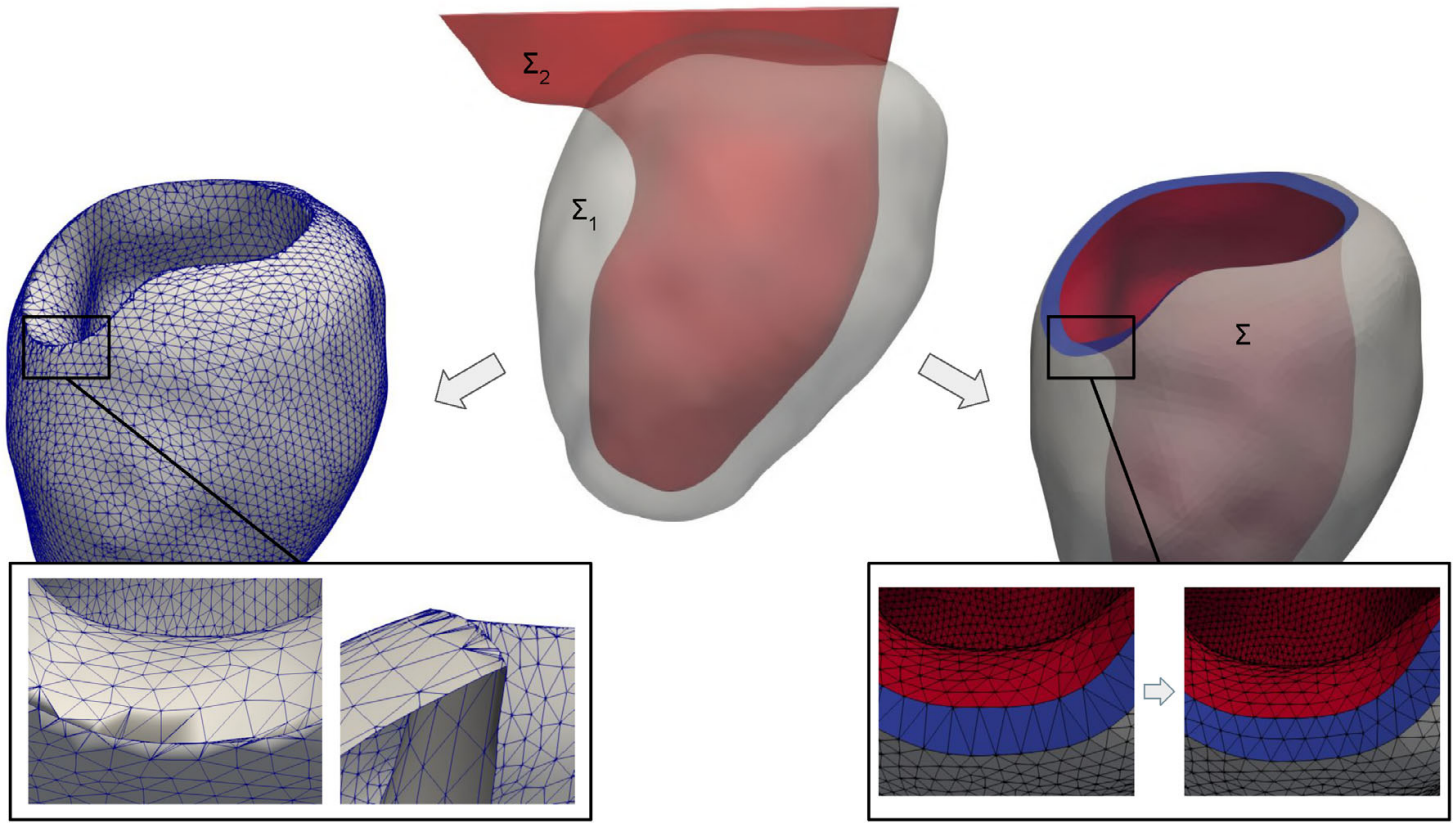
Comparison between the connection of two intersecting surfaces using classical boolean operations and the proposed *boolean‐connection* algorithm: on the center, the left‐ventricular epicardium (∑_1_) and endocardium (∑_2_) as delivered by the segmentation process; on the left, the output of a classical boolean difference operator ∑_1_ − ∑_2_ which produces irregular triangles and narrowed regions, as depicted in the zoomed box; on the right, the output (∑) of the same difference computed with the *boolean‐connection* algorithm which generates a smooth surface made of regular triangles and which also assigns the tags of the endocardium (red), the epicardium (gray) and the newly‐generated valvular annulus (blue)

Outputs of cardiac medical imaging segmentation can be intersecting surfaces. For instance, it is common to reconstruct the endocardium and the epicardium of the left ventricle separately. Since the volume occupied by blood is usually the part with more contrast in a cardiac image—either because of an injection of a contrast agent (like in CT‐scans) or because of specific acquisition protocol (like in cardiac MRI)—the endocardium is reconstructed as the external limit of the blood regions inside the heart. This typically produces a surface that includes a part of the left‐atrial endocardium and of the aortic root. On the contrary, the epicardium is typically reconstructed as a closed surface capped at the valvular annulus. An example of such kind of segmentation outputs is shown in Figure 4, center. In that case the left‐ventricle can be reconstructed by connecting the two surfaces at their intersection. This operation can be achieved with boolean operation between surfaces,^80^ in particular using the difference boolean operator. However, the output surface can be characterized by irregular triangles and narrowed regions on the intersection zone, as shown in the zoomed box of Figure 4, left. This kind of angular geometry makes the volumetric mesh generation challenging or even not possible without any additional processing. For these reasons, we propose a slightly different approach that generates as output a smoother and already tagged surface ready for volumetric mesh generation, as shown in Figure 4, right, for the aforementioned example.

##### Input, output, parameters and options

Two triangulated surfaces—named ∑_1_ and ∑_2_—intersecting in one or more closed lines are taken as input. Both ∑_1_ and ∑_2_ should be closed surfaces, otherwise an undesired output could be produced since the internally‐used signed distance is not robustly defined for open surfaces. Fortunately, polygonal surfaces generated by a medical image segmentation are usually closed. Another requirement is that both surfaces are characterized by outward normals, as it is common for polygonal closed surfaces. Our algorithm provides as output a unique closed surface ∑ defined as the difference, the union, or the intersection between ∑_1_ and ∑_2_. The two input surfaces are clipped at a user‐defined distance *ε* from their intersections and then connected using the *surface‐connection* algorithm. Optionally, the clip operation can be done only on a single input surface. Moreover, the final surface ∑ is remeshed—optionally only near the intersection zone—using a user‐defined constant mesh‐size *h*.

##### The *boolean‐connection* algorithm

1. On ∑_1_, compute the signed distance *d*_1_ from ∑_2_ and vice‐versa to obtain *d*_2_;
2. clip ∑_1_ and ∑_2_ at their intersection, that is, at the level where *d*_1_ = *d*_2_ = 0. Name the two resulting boundary rings Γ_1_ and Γ_2_. At this stage, ∑_1_ and ∑_2_ are split into two parts: $\sum_{1}^{+}$ and $\sum_{2}^{+}$ where *d*_1_ ≥ 0 and *d*_2_ ≥ 0, respectively; $\sum_{1}^{-}$ and $\sum_{2}^{-}$ where *d*_1_ ≤ 0 and *d*_2_ ≤ 0, respectively;
3. depending on the desired boolean operation, keep only the negative or the positive part of the two surfaces and name them as $\sum_{1}^{*}$ and $\sum_{2}^{*}$, respectively:
  - *difference*: $\sum_{1}^{*}=\sum_{1}^{+}$ and $\sum_{2}^{*}=\sum_{2}^{-}$;
  - *union*: $\sum_{1}^{*}=\sum_{1}^{+}$ and $\sum_{2}^{*}=\sum_{2}^{+}$;
  - *intersection*: $\sum_{1}^{*}=\sum_{1}^{-}$ and $\sum_{2}^{*}=\sum_{2}^{-}$;
4. on $\sum_{1}^{*}$ and $\sum_{2}^{*}$ compute the unsigned distances *D*_1_ and *D*_2_ from the boundary rings Γ_1_ and Γ_2_, respectively;
5. using the user‐defined parameter *ε*, clip $\sum_{1}^{*}$ and $\sum_{2}^{*}$ at the level where *D*_1_ = *D*_2_ = *ε* and keep the part where *D*_1_ ≥ *ε* and *D*_2_ ≥ *ε*, respectively. Optionally, this operation can be done only on $\sum_{1}^{*}$ or on $\sum_{2}^{*}$, to preserve the sharpness of the intersection;
6. remesh both $\sum_{1}^{*}$ and $\sum_{2}^{*}$ using the parameter *h* as constant mesh‐size, in order to generate better quality triangles at the level of the clip, where the generated polygons would be otherwise split into narrowed triangles. This remeshing can be optionally performed only on a 3 *h*‐wide “buffer‐zone,” in order to avoid remeshing those regions far from the intersection zone;
7. now $\sum_{1}^{*}$ and $\sum_{2}^{*}$ both have open boundaries near the intersection zone made of regular triangles. Thus, we can connect them by exploiting the *surface‐connection* algorithm (Section 2.1.1) generating the continuous conforming surface ∑. This operation also assigns specific tags to the three different regions of ∑ ($\sum_{1}^{*}$, $\sum_{2}^{*}$, and the connecting ring). If more than one boundary per‐surface are present, each boundary is automatically connected with the nearest one;
8. remesh also the connecting zone of the final surface ∑ with the constant mesh‐size *h*.

##### Discussion

In Figure 4 we show the result of the difference operator between the epicardium (∑_1_) and the endocardium (∑_2_) of the left‐ventricle. We compare the results of a traditional boolean algorithm^80^ (on the left) with the newly proposed *boolean‐connection* algorithm (on the right). Thanks to the clip at distance *ε* from the intersection ring, the output of our algorithm consists in a smoother surface, with no unrealistic narrowed regions at the valvular annulus. Moreover, the automatic generation of tags identifies particular regions like the valvular annulus (Figure 4, right, in blue) that cannot be easily reconstructed from medical images and that can be defined with a realistic thickness by suitably tuning the parameter *ε*. Finally, the integrated remeshing process generates a surface made of regular triangles, avoiding the creation of degenerate stretched elements. The output surface is now ready for the volumetric mesh generation, contrary to what would happen with traditional algorithms. Thus, the proposed algorithm can contribute to speed up the mesh generation process, especially when the focus is a patient‐specific single‐chamber model as the one shown in Figure 4. For other applications, however, a “smooth” connection can produce a worse result. For instance, this can happen when an artificial device—like the implantation of a cannula—orthogonally intersects a vessel or a cardiac chamber. In this case, the user can activate the option to perform the clip operation only on a single input surface (see step 5 of the algorithm). Indeed, this will produce the same sharp intersection provided by the traditional algorithm, but still taking advantage of the integrated remeshing procedure that generates a surface ready for the volumetric mesh generation.

Although we have limited our example to the difference operation, union and intersection operations may also be useful in the cardiac context. For instance, union can be used to connect the atrial endocardium with the ventricular endocardium when the segmentation produces two closed surfaces reconstructed from two different medical images.

#### 2.1.3 Connecting two surfaces through a deformation: the *harmonic‐connection* algorithm

##### Motivations

**FIGURE 5:**
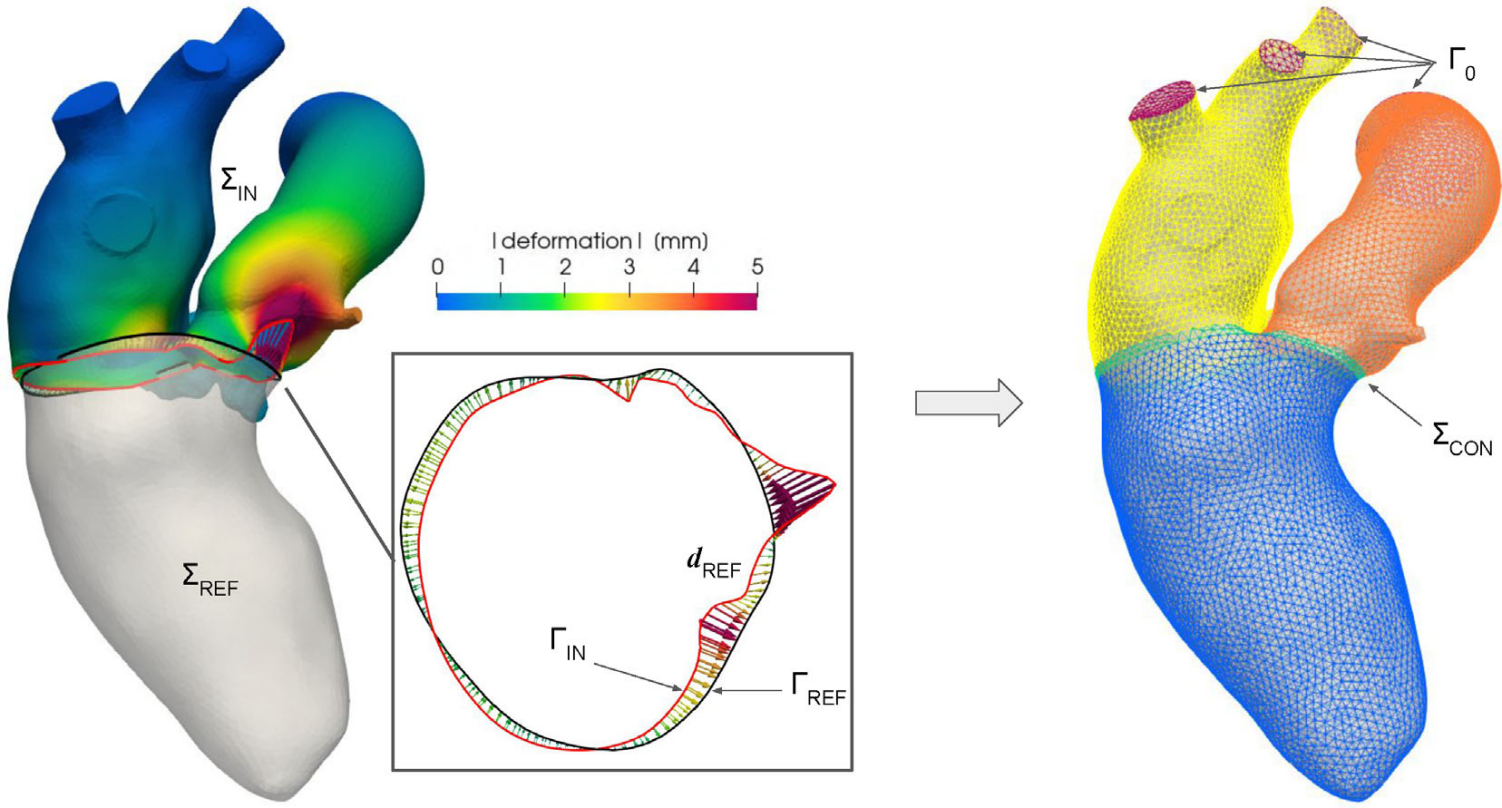
The *harmonic‐connection* algorithm in action: a template geometry ∑_IN_ of the left‐atrium and ascending aorta is deformed till its boundary ring Γ_IN_ (in red) matches the boundary ring Γ_REF_ (in black) of a patient‐specific left‐ventricle geometry ∑_REF_. On the left, the distance vector ***d***_REF_ between the two rings Γ_IN_ and Γ_REF_ and the resulting deformation obtained by harmonically extending ***d***_REF_ from Γ_IN_ to the whole surface ∑_IN_; on the right, the final continuous triangulated surface ∑ colored with its tags, where the purple ones represent the homogeneous Dirichlet boundaries Γ_0_ of the harmonic problem

The *surface‐connection* algorithm (Section 2.1.1) is used to connect two disconnected surfaces separated by a gap, creating a connecting triangulation between two boundary rings. However, in some applications we could be interested to attach a given surface to a reference surface, by deforming its boundary ring onto the reference one. In Figure 5, as an example of this situation, we show a case that may happen for example, for patient‐specific hemodynamic simulations,^28^ when only a part of the computational domain can be reconstructed from medical images: the left ventricle endocardium (in gray) is segmented from a short‐axis cine‐MRI—that in standard clinical exam typically captures only the ventricular geometry—while the remaining left‐heart comes from a template geometry, in this case the Zygote Solid Heart Model.^15^ In order to address this situation, we propose here a new algorithm that exploits the solution of a Laplace‐Beltrami equation.

##### Input, output, parameters and options

Our algorithm requires as input a surface ∑_IN_ and a reference surface ∑_REF_, both with at least a boundary ring Γ_IN_ ⊂ ∑_IN_ and Γ_REF_ ⊂ ∑_REF_. Note that the quality of the triangulation ∑_IN_ is important to guarantee a good numerical approximation of the Laplace‐Beltrami equation. The two surfaces can also be far from each other, since a rigid registration between the two open boundaries Γ_IN_ and Γ_REF_ can be performed at the beginning of the algorithm. As output, a unique continuous surface ∑ is created. Briefly, ∑ is obtained by deforming Γ_IN_ to match Γ_REF_ and then by harmonically extending the deformation on the whole ∑_IN_, as shown in the example of Figure 5. The deformed input surface ∑_IN_^DEF^ ⊂ ∑ can be optionally remeshed to recover, in case of large deformations, the regularity of the triangulation. User can also force a null deformation on a subset of ∑_IN_ selected through a set of tags $T\subset \sum_{\text{IN}}$. Thus, we define the deformation domain as $\sum_{*}=\sum_{\text{IN}}\setminus T.$ Both homogeneous Neumann or homogeneous Dirichlet conditions can be imposed at the free boundaries Γ = *∂*∑_*_∖Γ_IN_. In case of remeshing of the deformed surface ∑_IN_^DEF^, a parameter *h* identifies the desired constant mesh‐size. This parameter is also adopted to set the thickness of the buffer zone between ∑_IN_^DEF^ and ∑_REF_, created to guarantee the conformity of the final triangulation ∑.

##### The *harmonic‐connection* algorithm

1. Select Γ_IN_ ∈ ∑_IN_ and Γ_REF_ ∈ ∑_REF_, using a graphical interface if necessary;
2. optionally perform a rigid registration of Γ_IN_ on Γ_REF_ and apply the resulting rigid transformation *R* to the whole ∑_IN_. This step can be performed in two different ways:
  - by simply aligning the geometric centers of Γ_IN_ and Γ_REF_. In this case the resulting transformation *R* is just a translation;
  - by performing an Iterative Closest Points (ICP) algorithm^81^, ^82^ in order to minimize the average distance between the two rings. In this case *R* includes also a rotation;
3. on Γ_IN_, compute the distance vector ***d***_REF_ from Γ_REF_. An example of ***d***_REF_ between the two registered rings Γ_IN_ and Γ_REF_ is shown on the zoomed box of Figure 5, left;
4. set the deformation ***φ*** equal to zero on the user‐defined excluded tags T and define the deformation domain $\sum_{*}=\sum_{\text{IN}}\setminus T$. The boundary of the domain ∑_*_ is divided into three non‐overlapping sets *∂*∑_*_ = Γ_IN_ ∪Γ_0_ ∪Γ, where Γ_0_ is the homogeneous Dirichlet boundary between T and ∑_*_, while Γ = *∂*∑_*_∖{Γ_IN_ ∪Γ_0_} is the remaining part of the boundary where homogeneous Neumann conditions are assigned;
5. at this stage, if Γ ≠ *∅*, the user can optionally move the boundary rings of Γ into Γ_0_, to fix a null deformation also on them;
6. find the harmonic deformation ***φ*** by solving the following vectorial Laplace‐Beltrami equation using the finite element method with piecewise linear elements^83^, ^84^:
  (1) $$\left\{\begin{array}{ll} \Delta \varphi =0, & \text{in}\;\sum_{*}\\ \varphi =d_{\mathrm{REF}}, & \mathrm{on}\;\Gamma_{\text{IN}}\\ \varphi =0, & \mathrm{on}\;\Gamma_{0}\\ \nabla \varphi \cdot n=0, & \mathrm{on}\;\Gamma \end{array}\right..$$
  A prerequisite for the accurate solution of this surface‐PDE is that a good‐quality mesh must be available. If this is not the case, a preprocessing step can be performed by using the remeshing algorithm of *vmtk* in order to improve the quality of the triangulation ∑_IN_. At the end of this stage, the deformation ***φ*** is defined as a vector on each point of the triangulation ∑_IN_; an example is shown in Figure 5, left;
7. obtain ∑_IN_^DEF^ warping the surface ∑_IN_ according to the vector ***φ***. Note that, at this stage, ∑_IN_^DEF^ and ∑_REF_ are already perfectly adhering at their boundary rings, but their elements are non‐conforming;
8. on ∑_IN_^DEF^ compute the distance *d*_REF_ from Γ_REF_ and clip the part where *d*_REF_ ≤ *h*, creating a small gap between the two surfaces;
9. connect ∑_IN_^DEF^ and ∑_REF_ through a conforming triangulation ∑_CON_ using the *surface‐connection* algorithm (Section 2.1.1). The thin connection ring ∑_CON_ is automatically remeshed with a constant mesh‐size *h* and a specific tag is assigned to it. In this way, we obtain the final continuous triangulation ∑ = ∑_IN_^DEF^ ∪∑_CON_ ∪∑_REF_, as shown in Figure 5, right, where also the remeshed thin region ∑_CON_ can be appreciated;
10. optionally also the deformed part ∑_IN_^DEF^ can be remeshed with the user‐defined constant mesh‐size *h*.

##### Discussion

The main idea of the *harmonic‐connection* algorithm is to automatically attach one surface to another through a harmonic map, by exploiting the distance between two of their boundary rings. The final result depends on the chosen parameters. Since the template geometry ∑_IN_ is not generally located in the same region of the patient‐specific one ∑_REF_, the first choice is about the rigid registration to be performed: geometric center alignment versus ICP algorithm. The former is preferable when ∑_IN_ and ∑_REF_ are coherently oriented; this is not a rare case, since most of medical images are based on the sagittal‐coronal‐axial system of Reference 85. If no information is available about the orientation, the ICP algorithm produces a good initialization between the two rings Γ_IN_ and Γ_REF_, as shown in Figure 5, left. However, if more complex registration algorithms—for example, based on medical image registration—are necessary, users can perform this step using external tools and providing the already registered surfaces as input. The other important setting regards the boundary conditions. In the example reported in Figure 5 we assign a homogeneous Dirichlet condition both at the outlet of the ascending aorta and at the pulmonary veins, setting the excluded tags T as these regions (colored in purple) in order to properly define Γ_0_. Thus, this choice of T fixes the external part of the template geometry ∑_IN_. In other applications it could be preferable to leave one or more boundaries free to move rigidly according to the deformation, assigning a homogeneous Neumann condition on it.

#### 2.1.4 Modification of the structure thickness: The surface‐thickening algorithm

##### Motivations

When a patient‐specific geometry is reconstructed from medical images, some regions of the reconstructed surface—for instance the atria, or some regions of the right ventricle—can be unrealistically thin. This can happen because the thickness of these chambers is comparable with the resolution of the standard medical images. Furthermore, remeshing or creating a volumetric mesh of a very thin structure can be challenging or rather impossible. The proposed *surface‐thickening* algorithm aims at enlarging the structure where it is too thin, according to a user‐defined threshold. Optionally, also the reverse operation can be done, that is, decrease the thickness of a surface where it is larger than a user‐defined threshold.

##### Input, output, parameters and options

The input of our algorithm is a closed surface ∑_IN_ with a function *τ*(***x***)—that is, an array defined at all the points—representing the local thickness of the structure. We will present an ad‐hoc algorithm to compute the thickness of a cardiac chamber in Section 2.3.2. As output, the algorithm provides a closed surface ∑ characterized by a minimum thickness *σ* chosen by the user, obtained by enlarging ∑_IN_ where its original thickness is lower than *σ*. Moreover, a set of tags $T_{\mathrm{EXC}}$ can be excluded from the thickening and a scalar factor *α*—by default set equal to 1—can be multiplied by the deformation. Finally, an invert‐option can invert the behavior of the algorithm, making thinner all the parts of the input surface where *τ*(***x***) ≥ *σ*.

##### The *surface‐thickening* algorithm

1. define the vectorial function ***w*** to deform ∑_IN_ as a function of the excluded tags $T_{\mathrm{EXC}}$, the threshold *σ*, the scalar factor *α*, and the local outward normal ***n***:
  - if the *invert‐option* is not active:
    (2) $$w\left(x\right)=\left\{\begin{array}{ll} \frac{1}{2}\alpha \left(\sigma -\tau \left(x\right)\right)n, & \text{if}\;\tau \left(x\right)<\sigma \;\text{and}\;x\notin T_{\mathrm{EXC}}\\ 0, & \text{otherwise} \end{array}\right.;$$
  - else:
    (3) $$w\left(x\right)=\left\{\begin{array}{ll} \frac{1}{2}\alpha \left(\sigma -\tau \left(x\right)\right)n, & \text{if}\;\tau \left(x\right)>\sigma \;\text{and}\;x\notin T_{\mathrm{EXC}}\\ 0, & \text{otherwise} \end{array}\right..$$
    Note that the $\frac{1}{2}$ factor is necessary since the deformation acts both on the internal and the external surfaces;
2. warp all the points ***x*** ∈ ∑_IN_ according to the just defined function ***w***, obtaining the deformed surface ∑.

##### Discussion

**FIGURE 6:**
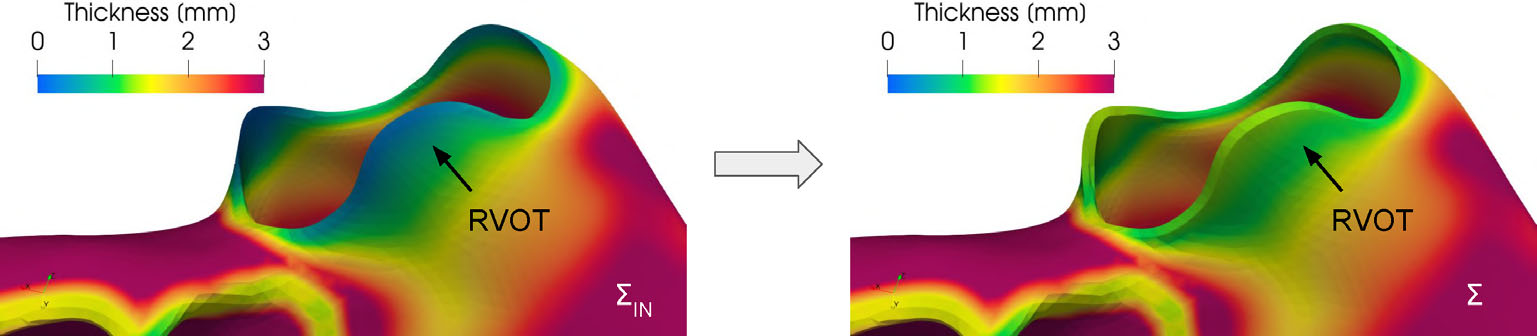
The *surface‐thickening* algorithm applied to an abnormally thin Right Ventricle Outflow Tract (RVOT): on the left, the thickness of the input surface ∑_IN_ characterized by a minimum value of 0.13 mm; on the right, the thickness of the enlarged output surface ∑ where a more physiological value of 1 mm is recovered

In Figure 6 we apply the *surface‐thickening* algorithm to a biventricular geometry in order to enlarge the surface in the region of the Right Ventricle Outflow Tract (RVOT), setting *σ* = 1 *mm* and *α* = 0.8. Despite the function ***w*** aiming to recover the desired minimum thickness *σ*, it acts in the surface normal direction. Thus, it is not guaranteed that, after the warping, the thickness is exactly equal to the desired value of *σ*. The scalar factor *α* can be exploited as a manual correction to achieve this purpose.

We remark also that, despite the fact that the default algorithm consists in moving all the points of the input surface where the local thickness is lower than a threshold *σ*, the set of tags $T_{\mathrm{EXC}}$ and the parameters *α* can be used to localize the algorithm only on a specific region of the surface or towards a specific direction. For instance, in the case of atrial septum—where the right and left epicardium are close to each other—the surface can be enlarged only towards the internal direction, in order to avoid triangles intersections at the epicardium. This can be achieved by setting $T_{\mathrm{EXC}}$ as the epicardium and *α* = 2, in order to create a deformation ***w*** that acts with a double magnitude only at the endocardium, still reaching the minimum deformed thickness *σ*.

### 2.2 Boundary tags definition: The *surface‐tagger* tool

**FIGURE 7:**
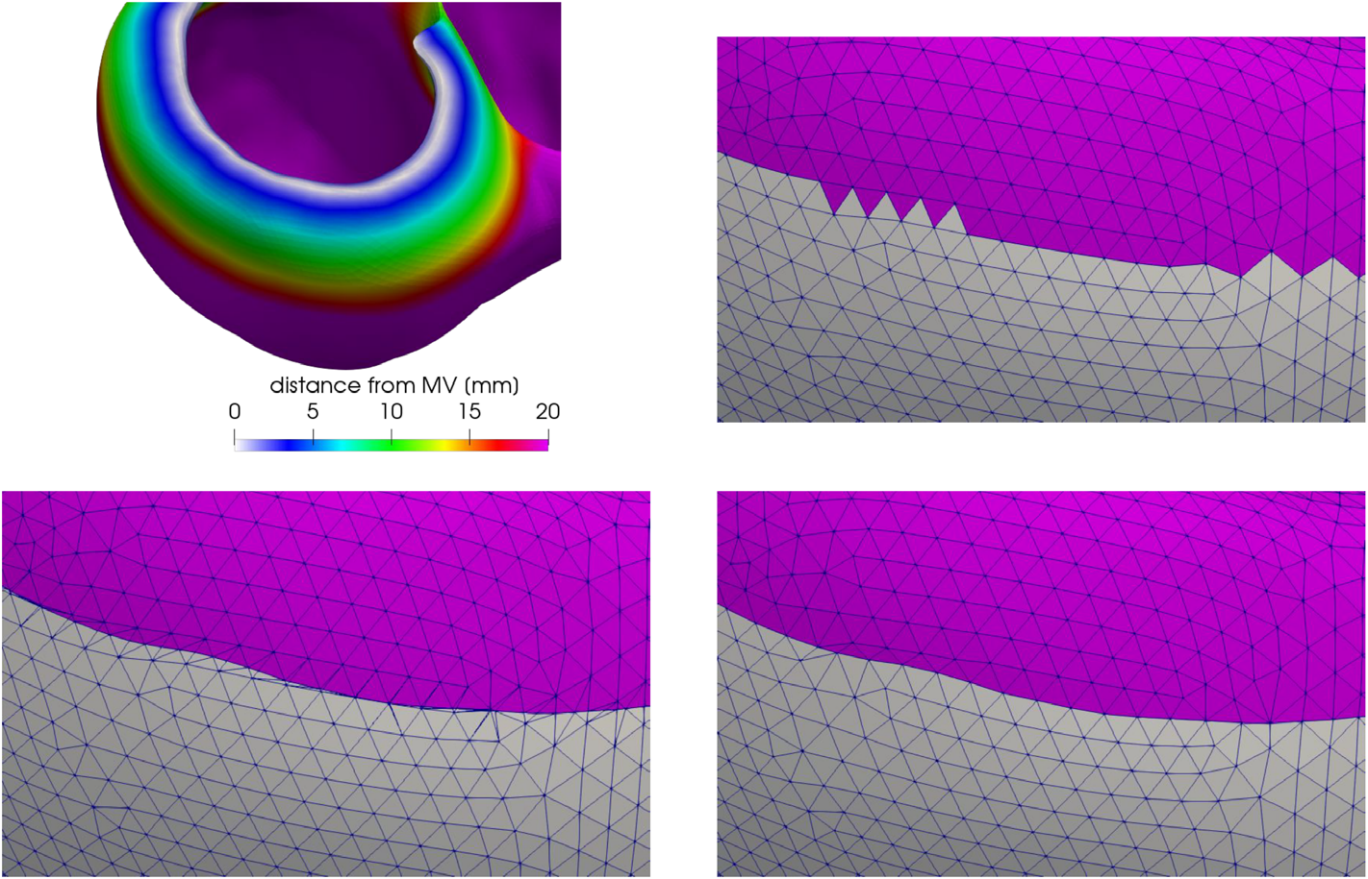
Comparison of the results of the three array‐based tagging algorithms included in the *surface‐tagger* tool, applied to a ventricular geometry on which the array representing the distance from the mitral annulus is defined (top‐left): on the top‐right the simple‐array algorithm; on the bottom‐left the *clip‐array* algorithm; on the bottom‐right the *harmonic‐array* algorithm. Details on the three algorithms can be found in Section 2.2

Assigning different tags to different regions of a surface is a crucial operation in a mesh generation pipeline. Indeed, tags can be exploited to impose boundary conditions or physical parameters, or to assign a specific mesh‐size to a part of the domain. In particular, boundaries of cardiac geometries are not clearly defined as sharp edges. For instance, on the ventricles, the limit between the endocardium and the epicardium lies on a smooth portion of the myocardial muscle, in proximity of the valvular annuli. A possibility in these cases is to generate the tags manually by exploiting a graphical interface. However, besides being time‐consuming, this procedure generates irregularly shaped tags, depending on the geometric distribution of the surface elements. In order to create more precise tags, we propose a tool that exploits significant functions defined on the surface, like, for instance, some distances from relevant objects. In practice, these functions are discrete arrays defined at each point of the polygonal surface. By selecting a cut‐off threshold, the values of these arrays can be used to assign two different tags to the elements. In detail, the proposed tool can perform this operation by choosing among three different algorithms, as illustrated in Figure 7, where we use the distance from the mitral valve annulus to tag a ventricular geometry:

1. The simple‐array algorithm assigns the tags without modifying the original triangles of the input surface. Thus, in most cases, it produces an irregular “zig‐zag” ring between tags, as illustrated in Figure 7, top‐right;
2. The *clip‐array* algorithm clips the elements exactly at the cut‐off value. In this way it produces precise tags, but distorted triangles, as shown in Figure 7, bottom‐left. Therefore, it is necessary to operate a surface remeshing that preserves the tags (this is possible with *vmtk*) to restore the regularity of the triangles;
3. The *harmonic‐array* algorithm produces regular and precise tags without creating any distorted triangle, as shown in Figure 7, bottom‐right. This is obtained by moving the points on the irregular “zig‐zag” ring created by the simple‐array algorithm till they lay onto the precise ring produced by the *clip‐array* algorithm. This movement is extended harmonically to the points of a surrounding buffer zone.

Finally, the *surface‐tagger* tool includes also two other algorithms for tagging a surface, that are not related with an array defined on it:

1. The connectivity algorithm, given an already tagged surface, assigns a different tag to each disconnected part of each tag;
2. The drawing algorithm allows the user to manually draw the tag using an interactive graphical interface.

##### Input, output, parameters and options

This tool needs as input a surface ∑_IN_, optionally with a set of tags already defined on it. As output, our algorithm generates a new tag *τ*_NEW_ on the output surface ∑. Users can select the desired tagging algorithm among the *simple‐array*, the *clip‐array*, the *harmonic‐array*, the connectivity, and the drawing. If an array‐based algorithm is selected, users must specify also the name of the array *f* to be used and its cut‐off threshold *σ*. Alternatively, the function *f* can be automatically computed as a signed or unsigned distance function from a reference surface ∑_REF_, provided by the user. A further parameter of the *harmonic‐array* algorithm is the radius *ρ* which represents the thickness of the buffer zone where the harmonic extension acts. Optionally, the algorithm can be limited to a subset of ∑, specifying a subset of tags T⊂∑ to be excluded.

##### The *surface‐tagger* tool

If the excluded tags T⊂∑ is a non‐empty set, the algorithms are performed on the surface ∑∖T and the subdomain T is merged to the final surface after the tagging operation. For the sake of simplicity, we detail hereafter the array‐based algorithms in the case of T=∅. The *connectivity* and the *drawing* algorithms are not discussed since they are based on existing *VTK* filters to select connected parts of a surface and to graphically interact with it, respectively.

**FIGURE 8:**
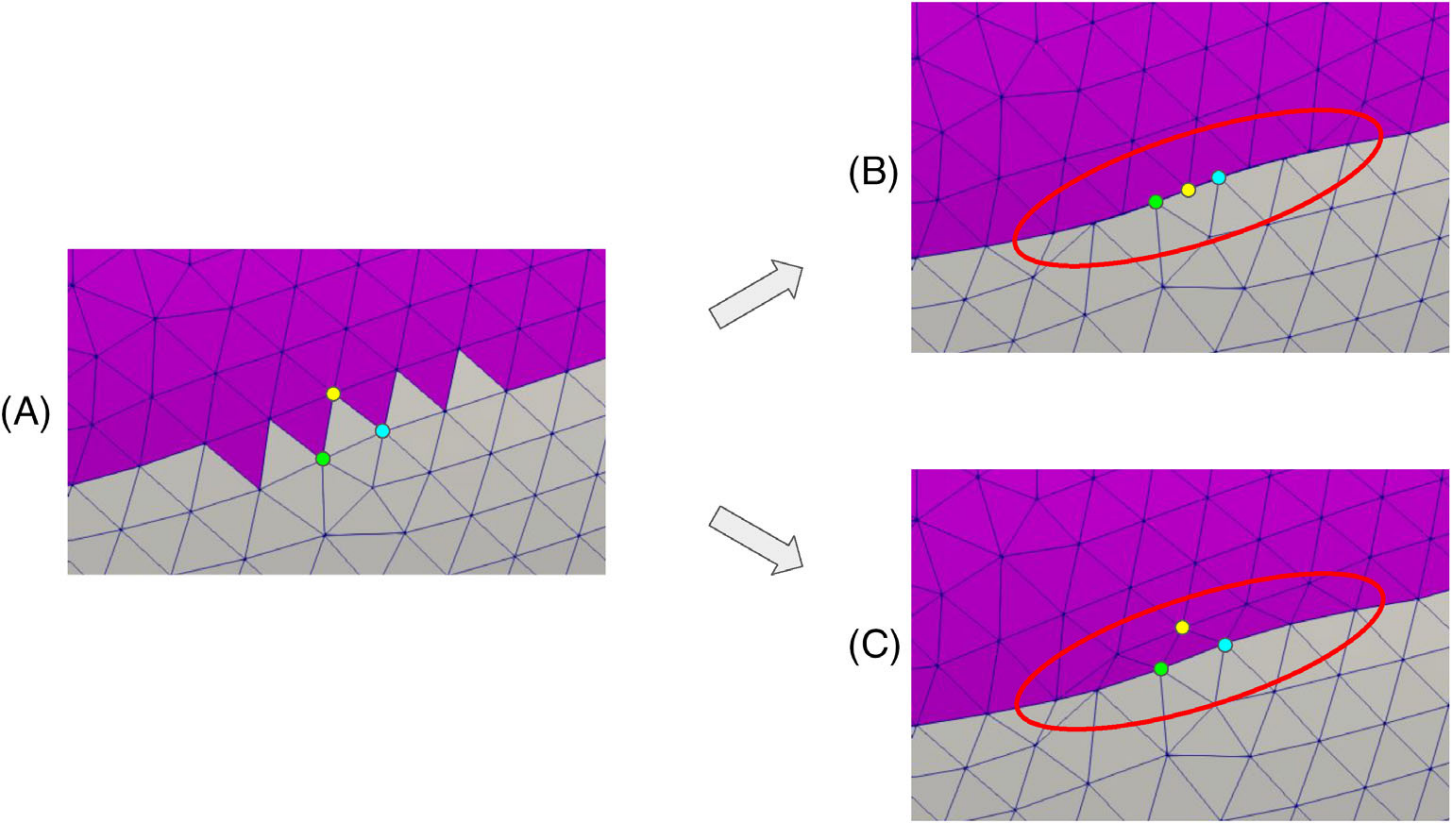
The *harmonic‐array* algorithm of the *surface‐tagger* tool in action: (A) initialization with the simple‐array algorithm; (B, C) the result obtained without (B) and with (C) the fixing‐point correction procedure. The vertices of a triangle are colored to follow their evolution; in red, the region where triangles collapse if not corrected

1. *Simple‐array*: given the surface ∑, the function *f*(***x***) defined on all the points *x* ∈∑, and the cut‐off value *σ*, the algorithm proceeds as follows:
  - Cycling on all the cells *c* of the polygonal surface ∑, evaluate the function *f* at the barycenter of the cell ***x***_***c***_;
  - If *f*(***x***_*c*_) ≤ *σ* assign the specific tag *τ*_NEW_, else leave the existing tag.
  In case of a non‐tagged input surface ∑, a different tag is also assigned to those cells where *f*(***x***_*c*_) > *σ*. Optionally the user can invert the behavior of the algorithm by assigning the tag *τ*_NEW_ to the cells where *f*(***x***_*c*_) ≥ *σ*. We refer to the irregular “zig‐zag” ring between the two tags created by this algorithm as Γ_ZIG_ (Figure 7, top‐right).
2. *clip‐array*:
  - clip the surface ∑_IN_ at the ring Γ_CLIP_ defined as the level where the function *f* is equal to *σ*, generating the two surface ∑_1_ and ∑_2_:
    (4) $$\Gamma_{\text{CLIP}}=\left\{x\in \sum_{\text{IN}}:f\left(x\right)=\sigma \right\};$$
    (5) $$\sum_{1}=\left\{x\in \sum_{\text{IN}}:f\left(x\right)\leq \sigma \right\};$$
    (6) $$\sum_{2}=\left\{x\in \sum_{\text{IN}}:f\left(x\right)>\sigma \right\}.$$
    The polygons arising on ∑_1_ and ∑_2_ after the clip are re‐triangulated splitting them in triangles (Figure 7, bottom‐left);
  - assign *τ*_NEW_ to ∑_1_. Also in this case, optionally, the behavior can be inverted assigning *τ*_NEW_ to ∑_2_;
3. *harmonic‐array*:
  - exploit the *clip‐array* algorithm to generate the Γ_CLIP_ ring;
  - exploit the simple‐array algorithm to generate the Γ_ZIG_ ring and initialize the ring Γ_Δ_ = Γ_ZIG_;
  - numbering the *N* points ***x***_*i*_ ∈Γ_ZIG_ with an index *i* = 0…*N* − 1 such that ***x***_*i*_ is connected to ***x***_*i*+1_, if three consecutive points {***x***_*i*_, ***x***_*i*+1_, ***x***_*i*+2_} belong to the same triangle *c* ∈∑_IN_, delete x_*_ = ***x***_*i*_ or x_*_ = ***x***_*i*+1_ from Γ_Δ_ connecting x_* − 1_ directly to x_*+1_. In particular, delete x_*_ = ***x***_*i*_ only if ***x***_*i* − 1_ ∈Γ_Δ_, that is, ***x***_*i* − 1_ was not already deleted. Otherwise, the point to be deleted is chosen as x_*_ = ***x***_*i*+1_. This step is necessary to avoid that some triangles successively collapse into lines, as shown in Figure 8. In particular, as an example, taking the triangle *c* as the one with the colored vertices, x_*_ would be the yellow vertex (i.e., x_*_ = ***x***_*i*+1_ in this case);
  - on ∑_IN_, compute the unsigned distance *d*_0_ from the ring Γ_Δ_ and, exploiting once again the *simple‐array* algorithm with *σ* = *ρ*, extract from ∑_IN_ the two “zig‐zag” rings Γ_0_ that define the homogeneous Dirichlet boundaries Γ_0_ delimiting the buffer zone;
  - on Γ_Δ_ compute the vectorial distance ***d***_Δ_ from the ring Γ_CLIP_;
  - defining ∑_*_ as the part of ∑_IN_ between the two rings of Γ_0_, solve the following Laplace‐Beltrami problem in ∑_*_, in order to find the deformation field ***φ***
    (7) $$\left\{\begin{array}{ll} \Delta \varphi =0, & \text{in}\;\sum_{*}\\ \varphi =d_{\Delta }, & \mathrm{on}\;\Gamma_{\Delta }\\ \varphi =0, & \mathrm{on}\;\Gamma_{0} \end{array}\right.$$
  - warp the surface ∑_IN_ according to the field ***φ***, obtaining the output surface ∑. At this stage the points of Γ_Δ_ lay on Γ_CLIP_, but the regularity of the triangles is preserved thanks to the previously executed step 3 (see Figure 8);
  - as the points of ∑ have been moved, the discrete array *f* defined on these new points must be updated according to the field ***φ***. If *f* is a distance, this update can be performed as a simple addition: *f* = *f* + |***φ***|. Otherwise, the field *f* must be recomputed on the deformed surface, by redoing the original computation;
  - finally, the tag *τ*_NEW_ can be assigned using the *simple‐array* algorithm with the updated field *f* and the cut‐off value *σ*

##### Discussion

The drawback of the *clip‐array* algorithm is that it must be followed by a surface remeshing algorithm in order to produce a surface ready for volumetric mesh generation. This may not be a problem when the user plans to define a non‐trivial tag‐dependent mesh‐size function, requiring a surface remeshing after the tagging operation. However, when this algorithm generates too many distorted triangles, the surface remeshing procedure could fail. Moreover, the remeshing is a very time‐consuming operation when dealing with complex geometries, like the one shown in Figure 1B. As an additional motivation, sometimes the non‐tagged geometry is already the desired one in terms of mesh‐size and mesh‐quality. This could, for instance, happen when the surface was already processed by a different mesh software that is unable to generate this kind of tags. In all these situations the *harmonic‐array* algorithm becomes very useful, since it shares all the advantages of the *clip‐array* algorithm, but not its disadvantages. Concerning the simple‐array algorithm, despite the irregular ring produced, this is the faster algorithm available and it can be used, for instance, when the need is just to assign a specific mesh‐size to a subregion for a successive remeshing. Similar considerations can be done for the drawing algorithm, that is also the unique possibility if no arrays are available. Finally, the connectivity algorithm is useful in some particular cases like, for instance, in order to distinguish between endocardium and epicardium after the individuation of the valvular rings. Indeed, in this case the valvular tags divide the ventricular geometry into disconnected parts. This algorithm automatically assigns a specific tag to each one of them.

We remark also that these algorithms can be performed on an already tagged surface and excluding a subset of tags from the new tag generation. This allows flexibility, since for each desired tag users can change the algorithm or array adopted.

### 2.3 Array processing and mesh‐size definition

#### 2.3.1 Extension of a field defined on a sub‐domain: The harmonic‐extension algorithm

##### Motivations

In several applications a scalar or vector field can be defined only on a subset T of a surface ∑. This situation can happen, for instance, for a deformation field reconstructed from dynamic medical images, for a cardiac fiber field reconstructed from DT‐MRI data, or even for some geometric quantities. A natural way to extend the field to the rest of the domain is to solve a Laplace‐Beltrami problem on the surface $\sum_{*}=\sum \setminus T$, using the values of the field at the boundary rings of the subregion T as Dirichlet boundary conditions of the problem. Clearly, the result of the extension depends on the boundary conditions imposed on the other boundaries $\Gamma_{*}=\partial \sum_{*}\setminus \partial T$, that is, all the boundaries of ∑_*_ that do not intersect the region where the original field is defined. Here, we present an algorithm to perform this harmonic extension automatically.

##### Input, output, parameters and options

On a subset T⊂∑ of the input surface a scalar or vector field $\varphi_{T}\left(x\right)$ is defined ∀x∈T. As usual, T can be identified by the user as a set of tags. On the domain $\sum_{*}=\sum \setminus T$ a further subregion $T_{*}\subset \sum_{*}$ can be selected by the user in order to impose a null Dirichlet value on it, similarly to the option available in the *harmonic‐connection* algorithm (Section 2.1.1). At the free boundaries Γ_*_, instead, both homogeneous Dirichlet or Neumann conditions can be imposed. Optionally, in order to increase flexibility, user can impose also non‐null constant values on these rings thanks to a graphical interface. Note that, like in the *harmonic‐connection* algorithm, also in this case the quality of the input triangulation is important in order to guarantee the quality of the numerical solution of the Laplace‐Beltrami equation.

##### The *harmonic‐extension* algorithm

1. Using the user‐defined set of tags T, define the domain of the harmonic extensions $\sum_{*}=\sum \setminus T$;
2. extract the common boundary ring of T and ∑_*_, defined as $\Gamma_{T}=\partial T\cap \partial \sum_{*}$, and initialize the Dirichlet and Neumann boundaries as empty domains, named Γ_DIR_ and Γ_NEU_, respectively;
3. if the optional set of tags $T_{*}\subset \sum_{*}\neq \emptyset$, reduce the extension domain to $\sum_{*}=\sum_{*}\setminus T_{*}$ and add the newly created boundary rings of ∑_*_ to the Dirichlet boundaries Γ_DIR_;
4. all the other boundaries of ∑_*_, if should they exist, can be optionally included into Γ_DIR_ by the user, otherwise they are assigned to Γ_NEU_. At this stage we have $\partial \sum_{*}=\Gamma_{T}\cup \Gamma_{\mathrm{DIR}}\cup \Gamma_{\mathrm{NEU}}$;
5. the Dirichlet data is initialized as a null function *g*(***x***) = 0, ∀***x*** ∈Γ_DIR_. Optionally, the function *g* can be modified through an interactive graphical interface, assigning a specific constant value at each ring;
6. solve the following Laplace‐Beltrami problem:
  (8) $$\left\{\begin{array}{ll} \Delta \varphi_{*}=0, & \mathrm{on}\;\sum_{*}\\ \varphi_{*}=\varphi_{T}, & \mathrm{on}\;\Gamma_{T}\\ \varphi_{*}=g, & \mathrm{on}\;\Gamma_{\mathrm{DIR}}\\ \nabla \varphi_{*}\cdot n=0, & \mathrm{on}\;\Gamma_{\mathrm{NEU}}. \end{array}\right.$$
  We remark that the function *φ* can be either a scalar or a vector field depending on whether the input field $\varphi_{T}$ is a scalar or a vector function;
7. finally, the extended field *φ* can be defined on the original surface ∑ as:
  (9) $$\varphi \left(x\right)=\left\{\begin{array}{ll} \varphi_{T}\left(x\right), & \forall x\in T\\ 0, & \forall x\in T_{*}\\ \varphi_{*}\left(x\right), & \forall x\in \sum_{*}. \end{array}\right.$$

##### Discussion

**FIGURE 9:**
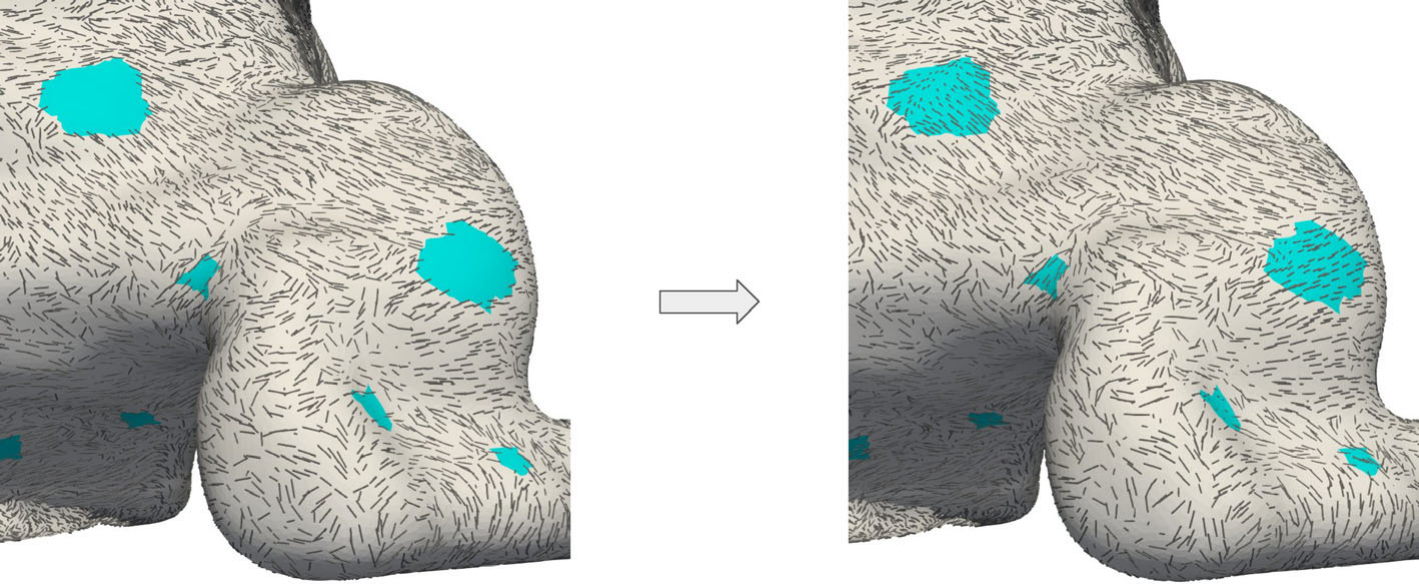
Recovering the left‐atrium fiber field on the epicardium using the harmonic‐extension algorithm: on the left, the field as reconstructed from DT‐MRI data where the regions with missing data are colored in light blue; on the right, the recover field. The data is taken from^86^

In Figure 9 we apply the algorithm to a fiber field on the left‐atrium epicardium reconstructed from DT‐MRI data, where the information is missing in many regions. The data is taken from Reference 86 and then processed to artificially create the missing regions. The harmonic‐extension algorithm succeeds in recovering the fibers on the missing regions (Figure 9, right), even in the case of sharp changes (see e.g., the uppermost region). Another possible example concerns the image‐based hemodynamics simulations in hybrid patient‐specific/template geometries (see Figure 5), where the deformation field reconstructed from dynamic medical images—for example, on the left‐ventricle—can be harmonically extended to the rest of the template domain.^28^

#### 2.3.2 Computing the thickness of a structure: The *surface‐thickness* algorithm

##### Motivations

The mesh‐size of the elements of a cardiac muscular mesh can be dependent from the thickness of the muscle. Here, we present an algorithm to compute the thickness of a structure that works also in case of biventricular geometries, where the presence of the septum makes however the definition of the thickness a little more involved.

##### Input, output, parameters and options

The algorithm takes as input a tagged surface ∑ where the internal and the external surfaces can be identified by the two sets of tags $T_{\mathrm{INT}1}\subset \sum$ and $T_{\mathrm{EXT}}\subset \sum$, respectively. In the specific case of a biventricular/biatrial geometry, the user has to pass separately two sets of tags $T_{\mathrm{INT}1}$ and $T_{\mathrm{INT}2}$ for the two disconnected internal surfaces which represent the left and the right endocardium. This allows to compute also the thickness of the ventricular/atrial septum.

##### The *surface‐thickness* algorithm

1. defining the domains $T_{\mathrm{INT}}=T_{\mathrm{INT}1}\cup T_{\mathrm{INT}2}$ and $T=T_{\mathrm{EXT}}\cup T_{\mathrm{INT}}$, compute the functions *D*_EXT_ and *D*_INT1_ on T as the unsigned distance from the surfaces $T_{\mathrm{EXT}}$ and $T_{\mathrm{INT}1}$, respectively.
2. if $T_{\mathrm{INT}2}\neq \emptyset$, compute also the two distances *D*_INT2_ and *D*_INT_, accordingly defined using the domain $T_{\mathrm{INT}2}$ and $T_{\mathrm{INT}}$, respectively.
3. ∀x∈T, compute the function *τ*(***x***) that represents the surface thickness distinguishing two cases:
  - if $T_{\mathrm{INT}2}=\emptyset$, then
    (10) $$\tau \left(x\right)=\max\left(D_{\mathrm{EXT}}\left(x\right),D_{\mathrm{INT}1}\left(x\right)\right);$$
  - if $T_{\mathrm{INT}2}\neq \emptyset$, then
    (11) $$\tau \left(x\right)=\max\left(\min\left(\max\left(D_{\mathrm{INT}1}\left(x\right),D_{\mathrm{INT}2}\left(x\right)\right),D_{\mathrm{EXT}}\left(x\right)\right),D_{\mathrm{INT}}\left(x\right)\right);$$
4. in order to define *τ* on the whole ∑, project the thickness function *τ* onto the remaining part of the input surface ∑∖T.

##### Discussion

Equation (11) combines all the computed unsigned distances in order to take into account the presence of the septum in a biventricular/biatrial geometry, where the thickness can be defined as the distance between the right and the left endocardium. However, the algorithm is not able to capture the thickness of thinner structures at the endocardium, like papillary muscles and trabeculae carneae. We remark also that the thickness on the part of the surface ∑∖T—that is, the valvular annulus—is defined by projecting the value computed at the boundaries of this region. Alternatively, in order to have a smoother function on ∑∖T, the thickness can be extended here using the harmonic‐extension algorithm (Section 2.3.1) as a further step of processing. Note that the thickness *τ* can be used directly as mesh‐size for smooth atrial or ventricular geometries (as the one shown in Figure 4), in order to obtain a volumetric mesh characterized by a constant number of elements from endocardium to epicardium. An example of thickness computed with this algorithm has already been shown in Figure 6.

#### 2.3.3 Mesh‐size computation: The surface‐mesh‐size tool

##### Motivations, input and output

In order to define a mesh‐size *h*(***x***) that depends on relevant geometric quantities, we propose here the *surface‐mesh‐size* tool which, given a tagged surface ∑, helps in the manipulation and combination of multiple arrays.

##### The *surface‐mesh‐size* tool

The tool assigns a specific mesh‐size function *h*(***x***) at each point of the tagged input surface ∑, according to one of the following algorithms chosen by the user:

1. the constant algorithm simply sets the mesh‐size as a constant positive value: *h*(***x***) = *γ*;
2. the array algorithm, given a function *f* on the input surface ∑, defines the mesh‐size as:
  (12) $$h\left(x\right)=\max\left\{m,\min\left\{\mathrm{\alpha f}{\left(x\right)}^{\beta }+\gamma ,M\right\}\right\},$$
  where the parameters of the expression are real numbers with the following default values: *α* = 1, *β* = 1, *γ* = 0, *m* = 0, and *M* =  + ∞. Hence, the mesh‐size is a function of the input array *f*, which depends on the multiplicative factor *α*, the exponent *β*, and the offset *γ*, and which is constrained in the interval [*m*, *M*];
3. the array‐combination algorithm behaves similarly to the array algorithm, but with the additional option of combining multiple input arrays *f*_*i*_. Indeed, supposing that the input is made of *N* functions *f*_*i*_, *i* = 1, …, *N*, a set of parameters {*α*_*i*_, *β*_*i*_, *γ*_*i*_, *m*_*i*_, *M*_*i*_} is assigned to each of them in order to define the corresponding mesh‐size function *h*_*i*_, according to Equation (12). Then, the final mesh‐size function is computed as:
  (13) $$h\left(x\right)=\min\left\{h_{1}\left(x\right),\ldots ,h_{N}\left(x\right)\right\}.$$

This algorithm allows to exploit more than one geometric quantities *f*_*i*_ computing the corresponding mesh‐size *h*_*i*_ and locally giving priority to the smaller *h*_*i*_.

Thus, depending on the chosen algorithm, the parameter of the tool can be a single scalar *γ*, a set of scalars {*α*, *β*, *γ*, *m*, *M*}, or N set of scalars {*α*_*i*_, *β*_*i*_, *γ*_*i*_, *m*_*i*_, *M*_*i*_}, *i* = 1, …, *N*. These parameters are used to initialize the mesh‐size function *h* on the whole surface ∑. If a mesh‐size *h* is already defined on ∑, the user can limit the computation of the new function *h* in a subset of tags T⊂∑. This operation can be optionally done also using a graphical interface which requires the user to provide the set of tags T where to modify the mesh‐size *h*, together with the chosen algorithm and the associated parameters. After each modification, the current mesh‐size is displayed; this allows the user to evaluate whether further changes are needed. Finally, together with the function *h*, the algorithm gives as output a smoother mesh‐size function $\hat{h}$ computed using the *vmtk* algorithm to smooth a discrete array, that is based on a local average on the values of nearby points. This provides a mesh‐size function without high local gradients and, consequently, to avoid a steep transition between small and large elements on the final volumetric mesh.

##### Discussion

**FIGURE 10:**
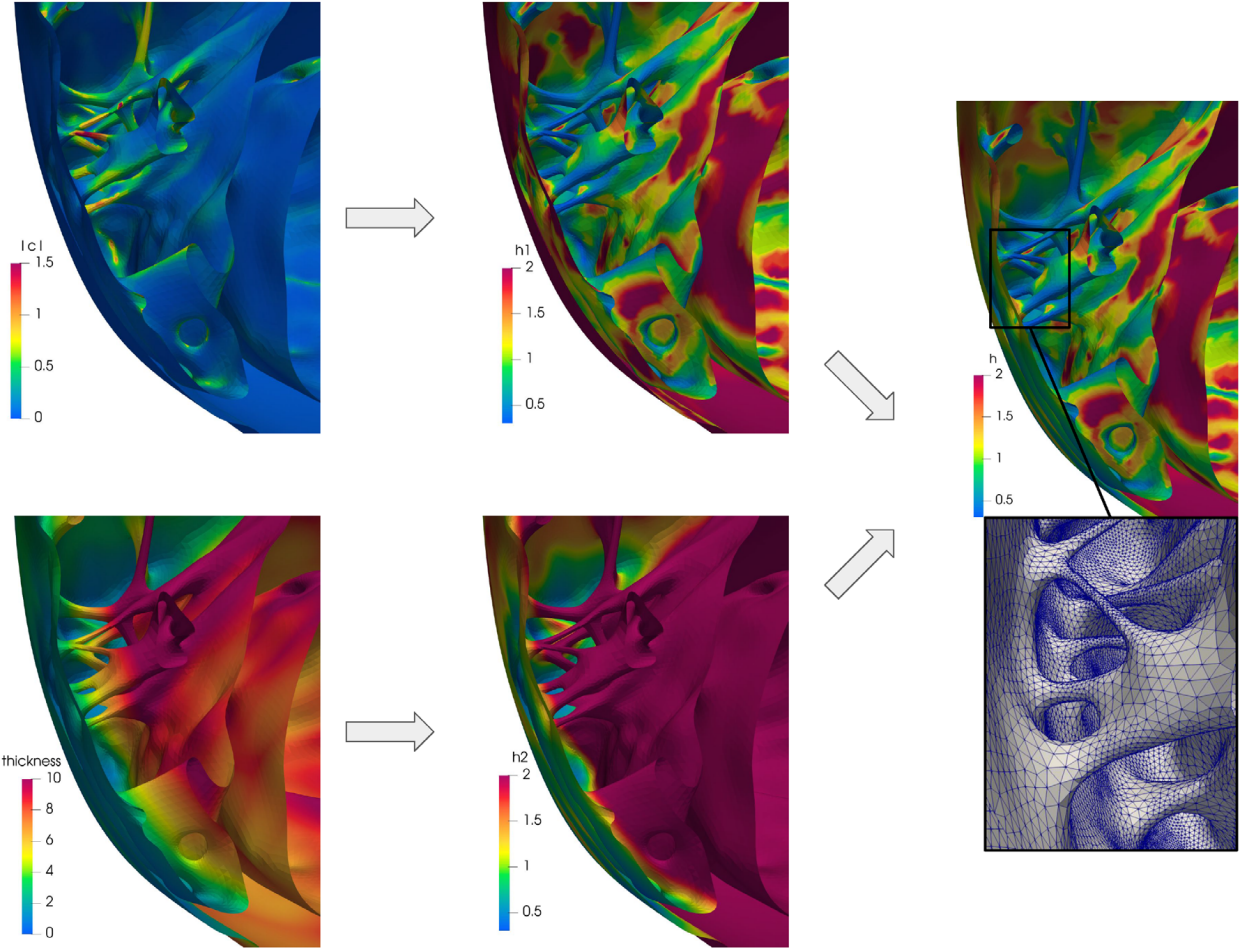
Computing the mesh‐size function *h* on the inferior part of the right ventricle: on the left, the magnitude of the mean curvature |*c*| (top) and the thickness (bottom); on the center, two mesh‐size functions *h*_1_ (top) and *h*_2_ (bottom) computed from the curvature and the thickness, respectively; on the right, the mesh‐size *h* obtained combining *h*_1_ and *h*_2_ and a zoomed detail of the related remeshed surface

In Figure 10 we show a detail of the right ventricle where the myocardium is very thin, the epicardium is smooth and the endocardium is very irregular because of the presence of the papillary muscles and the trabeculae carneae. This is a case in which the combination of more than one geometric quantities is necessary. In particular, we exploit the array‐combination algorithm selecting *f*_1_ as the mean curvature—computed using *vmtk* ^75^—and *f*_2_ as the myocardial thickness (Figure 10, left). The result shown on the right is obtained by fixing *α*_1_ = 0.3, *β*_1_ = −0.5, *α*_2_ = 0.5, *β*_2_ = 1, *γ*_1_ = *γ*_2_ = 0, *m*_1_ = *m*_2_ = 0.3, and *M*_1_ = *M*_2_ = 2. Thus, the final mesh‐size *h* on the one hand behaves as the inverse of the square root of the mean curvature and on the other hand constraints at least two elements per‐thickness. We remark that the combination of these two quantities through the minimum operator is a conservative way to define *h* (Figure 10, right). Indeed, in this example, using just the curvature would produce larger elements at the smooth epicardium (Figure 10, top‐center). Consequently, on the thinner region of the muscle the final mesh would be characterized by distorted elements that connect the large elements at the epicardium with the small ones at the endocardium. The combination of the curvature and the thickness overcomes this problem.

Automatic tetrahedral mesh generators that are able to remesh a surface according to the local curvature have been already proposed.^68^ However, the tool that we have just described is more suitable for the definition of mesh‐size functions in the complex cardiac geometries, thanks to the possibility of choosing a combination of an arbitrary number of quantities. Moreover, since no constraints are given on the input arrays, they can also be quantities computed from a numerical simulation on a coarser mesh, in order to define the mesh‐size as a function of local solution gradients. Finally, the possibility of defining a specific mesh‐size on each tag can be used when, for instance, specific geometric quantities can be associated to different regions or a particular region needs higher resolution. An example of this type will be discussed in Section 3.

### 2.4 Volumetric mesh processing

#### 2.4.1 Join two volumetric meshes: The mesh‐connection algorithm

##### Motivations

When dealing with multi‐chamber simulations, connecting two volumetric meshes is a necessary operation. For instance, for the mechanical model of the whole heart, the atria and the ventricles can be considered as separate volumetric meshes to be connected by a volumetric ring that represents the fibrotic tissue of the valvular annulus. This separation can also help to assign the correct mechanical property to each part of the model. Indeed, different tags can be assigned to each distinguished geometries in order to be able to associate this tag to chamber‐dependent parameters and constitutive law.^45^ Similar considerations can be done for electrophysiological models, where each chamber has specific electric property (e.g., conductivity, ionic model), while the valvular annulus must not conduct.

##### Input, output, parameters and options

**FIGURE 11:**
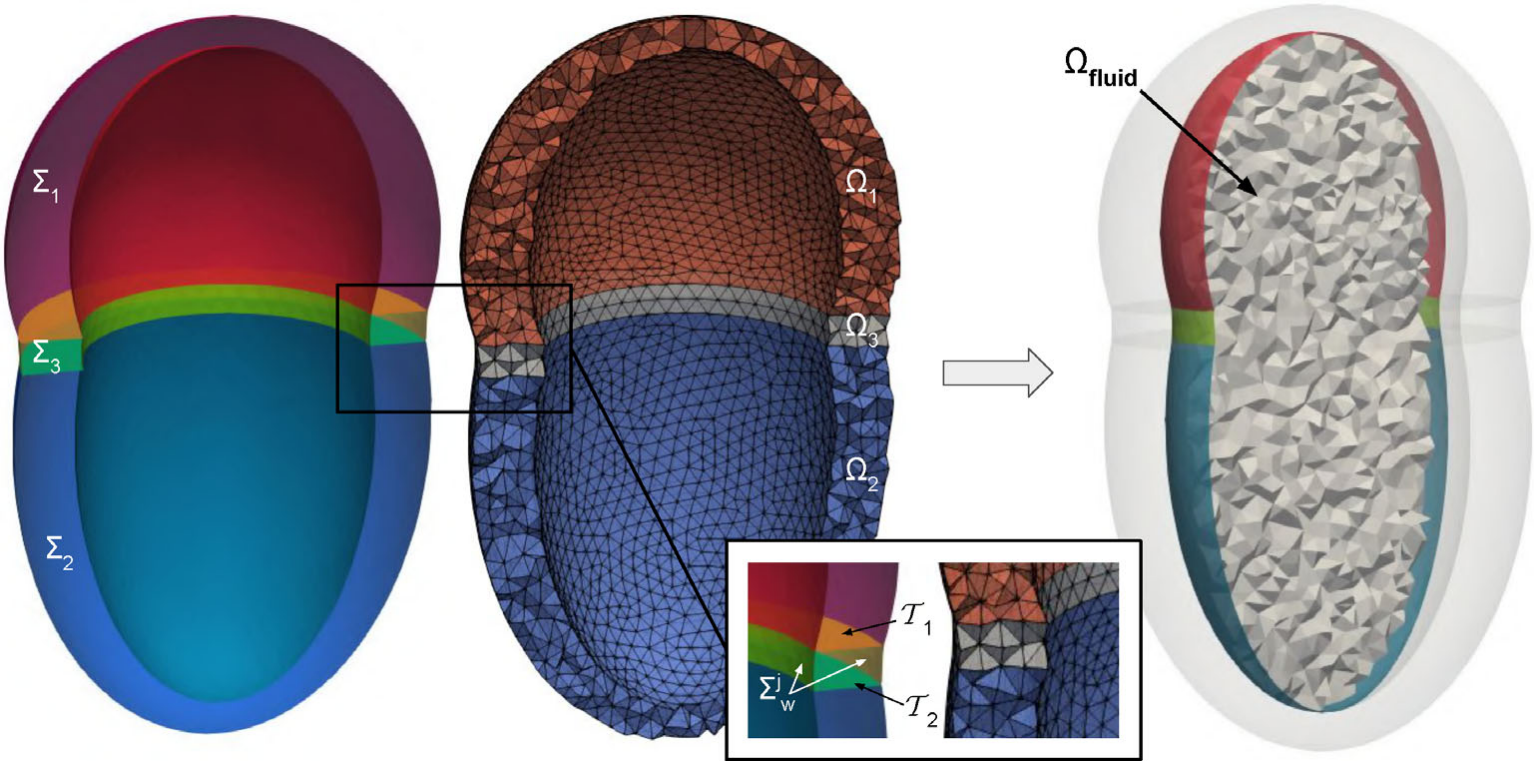
Connecting an idealized left atrium mesh Ω_1_ and an idealized left ventricle mesh Ω_2_ at their annulus ($T_{1}$ and $T_{2}$) using the *mesh‐connection* algorithm that automatically generates the connecting volumetric ring Ω_3_. The final mesh Ω = Ω_1_ ∪Ω_2_ ∪Ω_3_ preserves all the original surface tags and the algorithm assigns a specific tag to each of the three volumes and to the connection walls $\sum_{w}^{j}$. Each surface/volume tag is displayed with a specific color in the figure. On the right, the conforming fluid mesh Ω_fluid_ obtained from the internal surface tags is shown

With the above consideration in mind, we propose an algorithm that, given two tagged volumetric meshes Ω_1_ and Ω_2_, generates a third connecting mesh Ω_3_. As output, the three meshes are merged in a unique one Ω = Ω_1_ ∪Ω_2_ ∪Ω_3_ where the three parts are distinguishable thanks to volumetric tags. Moreover, all the original surface tags are maintained and specific tags are assigned to each new generated surface. Thus, naming the external surfaces of the two meshes ∑_1_ = *∂*Ω_1_ and ∑_2_ = *∂*Ω_2_, respectively, the regions on the two surfaces to be connected can be selected as two sets of tags: $T_{1}\subset \sum_{1}$ and $T_{2}\subset \sum_{2}$, respectively. Note also that, in order to be connectible, $T_{1}$ and $T_{2}$ need to be two topologically equivalent regions. Thus, they need to have the same number *n* of boundary rings. Finally, also the mesh‐size *h* of the connection mesh Ω_3_ can be set by the user. All the introduced notations are reported in Figure 11 where the connection between two meshes of two idealized cardiac chambers is shown.

##### The *mesh‐connection* algorithm

1. From the two input meshes Ω_1_ and Ω_2_, extract the regions to be connected: $T_{1}\subset \sum_{1}$ and $T_{2}\subset \sum_{2}$;
2. extract the *n* boundary rings of each of the two regions, named $\Gamma_{1}^{i}\subset T_{1}$ and $\Gamma_{2}^{i}\subset T_{2}$, respectively, where *i* = 1…*n*. Note that the two regions need to have the same number *n* of rings to be connectible;
3. at this stage, we aim to define the external surface of the connection mesh ∑_3_ = *∂*Ω_3_, that must be a closed surface. Thus, initializing $\sum_{3}=T_{1}\cup T_{2}$ and *j* = 1, while *j* ≤ *n* we proceed as follows:
  - exploiting the *surface‐connection* algorithm (Section 2.1.1), connect the boundary ring $\Gamma_{1}^{j}$ with one of the rings $\Gamma_{2}^{i}$, *i* = 1…*n*, producing the connection surface $\sum_{w}^{j}$, as shown in the zoomed box of Figure 11. The ring $\Gamma_{2}^{i}$ can be chosen either automatically—selecting the one at minimum average distance—or manually—through an interactive graphical interface;
  - assign a specific tag $\tau_{w}^{j}$ to the newly generated connection surface $\sum_{w}^{j}$;
  - update $\sum_{3}=\sum_{3}\cup \sum_{w}^{j}$ and *j* = *j* + 1.
  Note that, as required, ∑_3_ is a closed surface after this cycle;
4. remesh ∑_3_ only at the connection surfaces $\sum_{w}^{j}$, *j* = 1…*n*, using the user‐defined constant mesh‐size *h*. Note that, excluding the other tags from remeshing, the conformity between the surface ∑_3_ and the two input meshes Ω_1_ and Ω_2_ is maintained;
5. generate the volumetric mesh Ω_3_ using the standard algorithm of TetGen^62^;
6. merge the three conforming meshes Ω_1_, Ω_2_ and Ω_3_ in a unique mesh Ω, cleaning all the repeated points and cells at the interfaces $T_{1}$ and $T_{2}$.

##### Discussion

The *mesh‐connection* algorithm internally uses the *surface‐connection* algorithm (Section 2.1.1) in order to generate an intermediate volumetric mesh between two input meshes. This requires that the two regions to be connected on the two input meshes are topologically equivalent. For instance, two possibilities can be the connection of two circular boundaries—for example, the inlet/outlet of two fluid‐dynamic meshes—or the connection of two annular boundaries—for example, the valvular annulus of two cardiac chambers. The latter case shows the ability of the algorithm, given the single‐chamber meshes, to build a complete mesh of the four heart chambers by generating all the connecting meshes at the various valvular annuli. Moreover, the fact that the original surface tags are kept on the final mesh and that specific tags are assigned to each volume is of fundamental importance for multi‐chamber cardiac modeling,^45^ since it allows to assign different electro‐mechanical properties and boundary conditions to each part of the geometry.

Finally, this tagged 4‐chamber mesh can also be exploited to generate a conforming fluid‐dynamic mesh. Indeed, at this stage, it is sufficient to perform some classic steps of mesh generation using the *vmtk* library: the extraction of the internal surface of the left/right heart; the creation of the flow‐extensions at the boundary vessels, the optional creation of a boundary layer of elements, and the generation of the volumetric mesh. In this way, we can generate a couple of conforming meshes—that is, the fluid‐dynamic mesh in the blood domain (see Figure 11, right) and the multi‐chamber electro‐mechanical mesh in the muscular domain (Figure 11, center)—that can be used for the electro‐mechano‐fluid simulations of the whole cardiac function.

#### 2.4.2 Local refinement: The *mesh‐refinement* algorithm

##### Motivations

In some applications, after the volumetric mesh generation, an a priori mesh refinement in an internal subdomain could be necessary,^87^, ^88^, ^89^ because of several reasons: for instance, the computation of accurate quantities on a specific part of the domain or the necessity of a higher resolution in a numerically challenging part of the domain. Here, we present a simple algorithm to perform this kind of local refinement.

##### Input, output, parameters and options

The algorithm takes as input a tagged mesh Ω_IN_ with a function *f* defined on its points—for example, a distance from a region of interest—to be used for the local refinement. The output mesh Ω is refined according to the sizing function *h*(***x***) = max{*m*, *αf*(***x***)^*β*^}, where *α* and *β* are two positive real numbers, and *m* represents the minimum allowed mesh‐size. In practice, naming *h*_IN_(***x***) the local mesh‐size of Ω_IN_, the refinement will modify only those elements where *h*_IN_(***x***) ≥ *h*(***x***).

##### The *mesh‐refinement* algorithm

1. Compute the refinement sizing function *h*(***x***) = max{*m*, *αf*(***x***)^*β*^} for all the points ***x*** ∈Ω_IN_, depending on the parameters chosen by the user;
2. refine the mesh Ω_IN_ in all the cells where *h*_IN_(***x***) ≥ *h*(***x***) in order to reach the target mesh‐size function *h*(***x***), obtaining the final mesh Ω. The possibility of refining only the elements where a user‐defined sizing function is lower than the actual mesh‐size is included in *TetGen*, where a constrained Delaunay refinement algorithm for adaptive quality tetrahedral mesh generation is implemented^62^;
3. in order to maintain the original tags, project all the volumetric and surface tags from Ω_IN_ into Ω. To carry out this operation, for each cell *c* ∈Ω with ***x***_*C*_ its barycenter, it is sufficient to find the cell *c*_IN_ ∈Ω_IN_ such that ***x***_*C*_ ⊂ *c*_IN_ and assign its tag *t*_IN_ to the cell *c*.

##### Discussion

**FIGURE 12:**
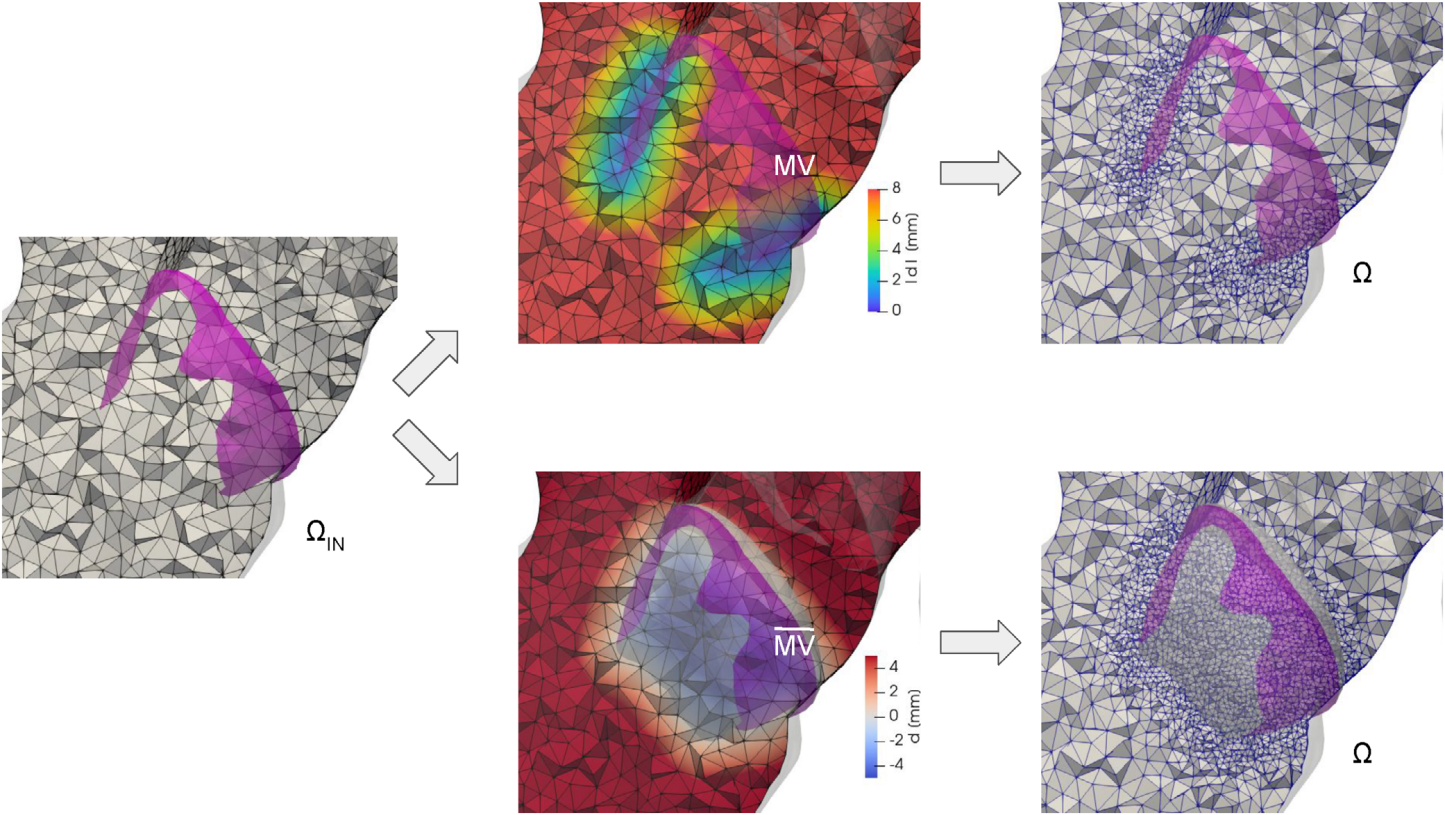
Two ways to perform a local mesh refinement on a fluid‐dynamic mesh of the left heart near the mitral valve: on the top, we exploit the unsigned distance |*d*| from the open leaflets (MV) in order to refine the mesh only near them; on the bottom, we use the signed distance *d* from a smoothly capped valve ($\bar{\mathrm{MV}}$) in order to refine the whole region inside the leaflets

In Figure 12 we show two different results of this algorithm applied to the local refinement of a fluid‐dynamic mesh of the left heart near the mitral valve. In both cases we set *α* = 0.2, *β* = 1, and *m* = 0.5. However, in the first case (on the top) *f* is the unsigned distance from the valve and it is exploited to obtain a mesh refined only near the leaflets. On the contrary, in the second case (on the bottom) *f* is taken as the signed distance from a capped version of the mitral valve. In this way *f* assumes negative values in all the elements inside this closed surface resulting on a refined mesh where the minimum mesh‐size *m* is assigned to all these elements. The former can be chosen if the valve movement is not taken into account by the numerical model considered, while the latter would be preferable to follow the valve deformation during a heartbeat. In both cases a smooth transition from the smaller to the larger elements can be appreciated. This kind of refinements is necessary when the valve is treated as an immersed surface in a fluid‐dynamics model^46^ without the necessity of using a conforming mesh with its surface. Indeed, in this cases the valve can be modeled as an implicit surface without the necessity of generating a fluid mesh conforming with its leaflets.^25^, ^28^

#### 2.4.3 Transforming a tetrahedral mesh into a hexahedral one: The *tet‐hex* algorithm

##### Motivations

Until now we have presented algorithms for surfaces made of triangles or meshes made of tetrahedral. As an alternative, hexahedral meshes could be used. In principle, they provide higher accuracy and reduced computational costs.^90^ However, their application in patient‐specific computational cardiovascular studies is challenging due to the complexity of the geometry and to the historical lack of automatic algorithms to generate volumetric elements.^90^ Recently, algorithms that produced a hexahedral mesh from a triangular surface have been proposed and used in the cardiac context.^43^ However, the generation of hexahedral meshes usually needs lots of user interactions, performed in ad‐hoc commercial softwares and resulting in a very manpower‐consuming operation that could also fail in complex cardiac geometries. Alternatively, the usage of a template mesh model to be adapted to the various patient‐specific geometries can be considered.^39^ However, also this strategy cannot be applied to complex detailed geometries. In this context, an always‐successful strategy is the generation of such kind of meshes simply dividing into hexahedra the elements of a tetrahedral mesh, resulting in a finer unstructured mesh. Here, we adopt this strategy by proposing an algorithm integrated in our pipelines to generate a hexahedral mesh from a tetrahedral one, maintaining the same surface and volume tags. We also propose a possible strategy to minimize the final distortion of the elements by exploiting the *Refine‐By‐Splitting* (RBS) algorithm (i.e., the refinement of a hexahedral mesh by splitting each hexahedron into eight hexahedra, halving the original mesh‐size).

##### Input, output, parameters and options

Given—as input—a tagged mesh Ω_TET_ made of tetrahedral inside the volume and of triangles at the boundaries, the algorithm produces—as output—the corresponding mesh Ω_HEX_ made of hexahedra and quads, respectively. Optionally, the resulting elements can be further refined through an arbitrary number *n*_RBS_ of RBS iterations. Tags of the input mesh Ω_TET_ are preserved on the output mesh Ω_HEX_.

##### The *tet‐hex* algorithm

**FIGURE 13:**
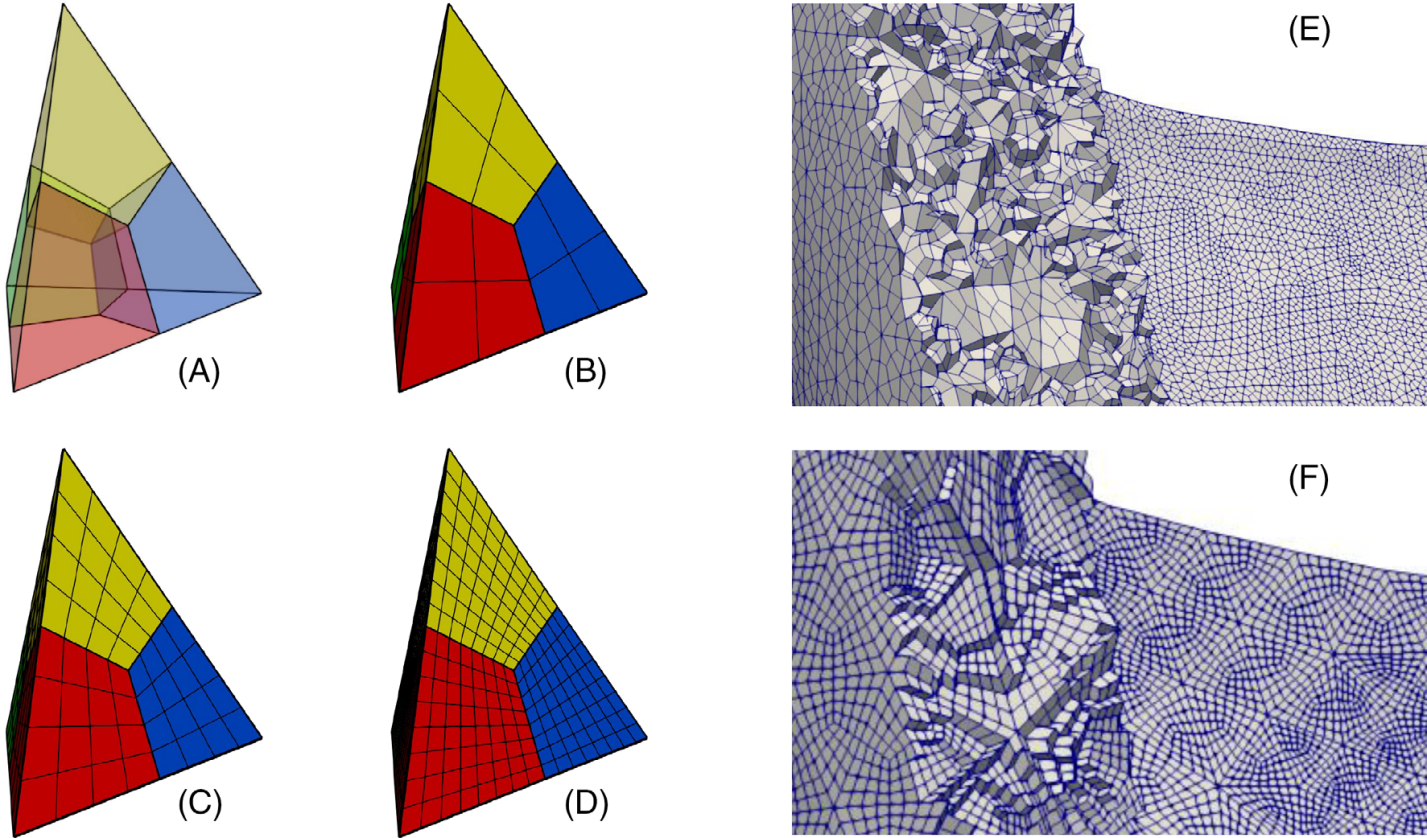
Transforming a tetrahedral mesh into a hexahedral one. On the left, the division of a single tetrahedron into four hexahedra (A) and three successive iterations (B–D) of the Refine‐By‐Splitting (RBS) procedure. On the right, the internal view of a left‐ventricle hexahedral mesh generated from a finer tetrahedral mesh with zero RBS (E) and from a coarser tetrahedral mesh with two RBS (F), respectively

Output elements are obtained dividing each triangle into three quads and each tetrahedron into four hexahedra, as shown in Figure 13A. More in detail, in order to obtain the quads from a triangle, its vertices, its barycenter, and the mid‐points of each edge are considered. Each quad is obtained by connecting each vertex with the two neighbor mid‐points and with the barycenter. Similarly, starting from a tetrahedron, the hexahedra are obtained by first dividing each face into three quads, then creating the four hexahedra using also the barycenter of the tetrahedron. Note also that, in practice, when defining an element it is important to consider the order of insertion of the points, in order to ensure an outward normal. The resulting quads and hexahedra can be iteratively split into four quads and eight hexahedra, respectively, through the RBS iterations, as shown in Figure 13B–D. Once again it is sufficient to consider the mid‐points of each edge, the barycenter of each face, and the barycenter of each hexahedron. During the definition of a new element the tag of the original element is preserved.

##### Discussion

The quality of the output hexahedral mesh depends on the quality of the input tetrahedral mesh. However, also in the case of a very regular tetrahedral mesh, the generated hexahedral elements are slightly distorted if compared with the ones of a regular structured mesh. This distortion decreases when performing additional RBS iterations. Indeed, in Figure 13, right, we compare two hexahedral meshes of a left‐ventricle geometry with similar average mesh‐size: the former (e) is obtained without RBS iterations from a finer tetrahedral mesh, while the latter (f) with two RBS iterations from a coarser tetrahedral mesh. It is evident that the latter is made of less distorted elements. Thus, a possible strategy is to start from the coarsest tetrahedral mesh possible, yet coherent with the constraint of geometric precision, and generate the corresponding hexahedral mesh reaching the desired mesh‐size by taking advantage of the maximum number of RBS iterations. The *tet‐hex* algorithm has the great advantage of always producing automatically a hexahedral mesh. Moreover, being implemented in the same context of the other algorithms presented in this paper, it can be easily integrated in a pipeline as final step. The other side of the coin is that, when dealing with very complex cardiac geometries, in order to accurately describe them the number of tetrahedral elements could already be very high. Consequently, the resulting hexahedral mesh could be too computationally demanding, considering that both the number of elements and the number of points grow significantly after the application of the algorithm. In conclusion, direct hex meshing methods are difficult, but preferable. However, considering the difficulties on producing native hexahedral meshes on complex cardiac geometries, this automatic algorithm represents a valid alternative in the cardiac context.

## 3 EXAMPLES OF MESH GENERATION PIPELINES

In this section, we show some examples of full cardiac mesh generation pipelines. In particular, in Section 3.1 we summarize how to integrate the proposed algorithms into few‐steps pipelines, depending on the cardiac application. Then, we present two more complex cases: the generation of a muscular mesh of a fully‐detailed ventricular geometry (Section 3.2) and the generation of a CFD mesh of the left‐heart (Section 3.3). These are only two examples among all the possible combinations, shown to make it clear how algorithms can be combined together. In Section 3.4, we conclude by showing some numerical studies performed on computational meshes generated using the algorithms proposed in this paper and related to the simulations of different processes of the cardiac function.

### 3.1 Some examples of combinations of new algorithms in few‐steps pipelines

A common occurrence is the generation of a patient‐specific single‐chamber mesh starting from the smooth segmentations of the epicardium and the endocardium. In this case the *boolean‐connection* algorithm (Section 2.1.2) automatically generates the external surface of the muscular geometry creating also three specific tags for the endocardium, the epicardium, and the valvular ring (see Figure 4) and remeshing the surface by a user‐specified constant mesh‐size. As an additional step, if we aim at generating a mesh with a constant number of elements per‐thickness, the thickness of the muscle can be computed using the *surface‐thickness* algorithm (Section 2.3.2), and the output can be optionally modified using the *surface‐thickening* algorithm (Section 2.1.4, see also Figure 6). Then, the mesh‐size can be defined as a function of the thickness using the *surface‐mesh‐size* tool (Section 2.3.3, see also Figure 10, top‐left and top‐center, for an example of such kind of mesh‐size function). Finally, the volumetric mesh is generated propagating onto the volumetric elements this mesh‐size using *TetGen*.^62^

If we are interested in generating a multi‐chamber mesh, once the single‐chamber meshes have been generated they can be connected with each other using the *mesh‐connection* algorithm (Section 2.4.1, Figure 11). In this case, also the conforming fluid‐dynamics mesh can be easily generated starting from the internal surfaces of the multi‐chamber mesh (see Figure 11, right).

A different case occurs when a patient‐specific surface describes only a part of the domain of interest of a numerical simulation. In this case the *harmonic‐connection* algorithm (Section 2.1.3, see also Figure 5) can be used to connect the patient‐specific geometry to a template geometry of the missing part of the domain. In this context, a scalar or vector field defined on the patient‐specific domain can be extended on the template domain using the *harmonic‐extension* algorithm (Section 2.3.1). Then, boundary tags can be created using the *surface‐tagger* tool (Section 2.2), the desired mesh‐size can be defined using the *surface‐mesh‐size* tool (Section 2.3.3), and the volumetric mesh can be generated accordingly. We also remark that additional steps of processing that can be applied to any kind of volumetric meshes are the *mesh‐refinement* algorithm (Section 2.4.2, Figure 12) and the *tet‐hex* algorithm (Section 2.4.3, Figure 13).

The aforementioned examples represent only some of the many possibilities that the combination of the proposed algorithms offers. Depending on the specific applications at hand, more complex pipelines can be built, as in the examples shown in the next two sections.

### 3.2 Generation of a fully‐detailed ventricular muscular mesh

We describe a complete pipeline for the generation of the volumetric mesh of the myocardium of a fully‐detailed biventricular geometry included in the *Zygote Solid 3D Heart Model*,^15^ a CAD geometry representing an average healthy heart. This geometry was reconstructed from high resolution CT‐scans of an healthy middle‐age male and scaled in order to reach the average size of an healthy heart.^15^ All the papillary muscles and trabeculae carneae are included in the geometry, making this case a challenging example of mesh generation to test our algorithms. In this pipeline we mainly exploit the *surface‐tagger* tool (Section 2.2) and the *surface‐mesh‐size* (Section 2.3.3) tool by showing how they can be used repeatedly to generate a tagged‐mesh of such a complex cardiac geometry.

**FIGURE 14:**
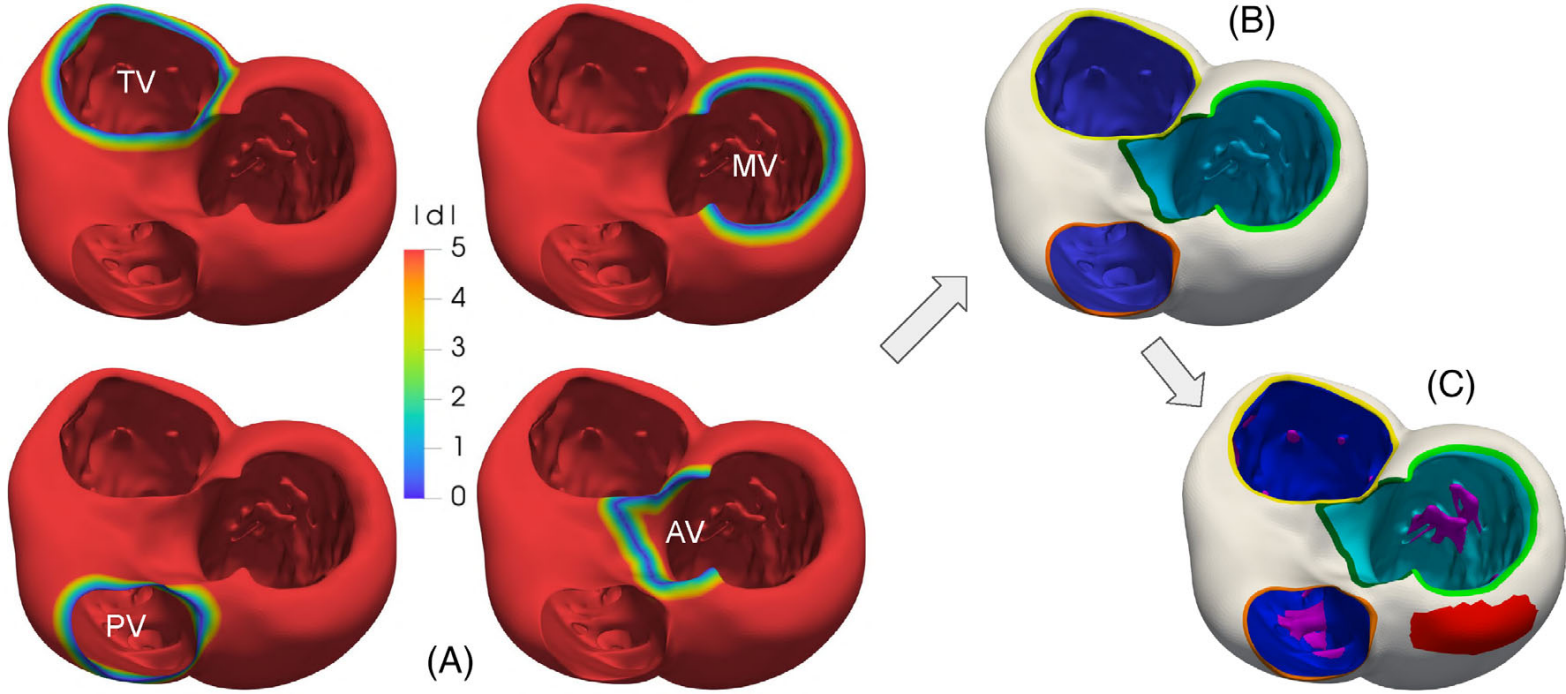
Tagging procedure for a biventricular geometry: (A) distances from the four valvular rings; (B) resulting tags of the four valvular annulus (TV in yellow, MV in pale green, PV in orange, AV in dark green), the epicardium (in gray), and the right and left endocardium (in blue and light blue, respectively), obtained using the *surface‐tagger* tool, *clip‐array* algorithm; (C) additional tags at the papillary muscles and trabeculae carneae (in violet), and at and a specific region of the left‐ventricular base (in red), generated using the *surface‐tagger* tool, array and drawing algorithms, respectively

First of all, we aim to precisely tag all the components of the biventricular geometry: the endocardium, the epicardium, and the four valvular rings. The latter are not identifiable as sharp edges on such kind of geometries. Thus, we have to exploit some suitable functions defined on the surface to generate these tags. From the *Zygote* model we can extract the valvular rings directly from the surfaces of the cardiac valves. Indeed, the Mitral Valve (MV), the Tricuspid Valve (TV), the Aortic Valve (AV), and the Pulmonary Valve (PV) are available as distinguished files in this CAD model. Hence, their rings can be used as reference data to compute on the myocardial surface the unsigned distances from them. In case the ventricular geometry was reconstructed from a routine medical image, this kind of data should be extracted directly by exploiting image processing tools. The resulting unsigned distances *d*_MV_, *d*_TV_, *d*_AV_, and *d*_PV_ from the four valvular rings are shown in Figure 14A. Exploiting these distances, the *surface‐tagger* tool can be used as follows:

1. for *i* = {MV, AV, TV, PV}, use the *clip‐array* algorithm fixing the cut‐off value *σ* = 1 *mm*. In this way, a specific tag is generated for each valvular annulus;
2. at this stage, the valvular rings are dividing the rest of the ventricular surfaces into three disconnected parts. Thus, we use the *connectivity* algorithm to generate three specific tags for the left‐ventricular endocardium, the right‐ventricular endocardium, and the epicardium.

The resulting tags are shown in Figure 14B. Note that, in this case we use the *clip‐array* algorithm instead of the *harmonic‐array* algorithm because, despite distorted triangles are generated at the boundaries of the tags, we plan to remesh the surface according to a newly defined mesh‐size function. In case the surface to be tagged is already remeshed with the desired mesh‐size (for instance with an external software) the *harmonic‐array* algorithm should be considered, since it produces both regular triangles and precise tags, as discussed in Section 2.2.

In order to define the mesh‐size function *h* on the surface, we proceed similarly to the example shown in Section 2.3.3 (Figure 10, but here we also want to explore the feature of defining different mesh‐size functions in different regions. For this purpose, we generate additional tags on the papillary muscles and on a part of the base of the left‐ventricle by proceeding as follows:

1. on the whole surface, compute the distance *d*_ENDO_ from a smoothed version of the left and right ventricular endocardium. Note that, in our case, this smooth endocardium can be obtained with standard processing on the original detailed CAD model. Conversely, in case of a geometry coming from a medical image processing, obtaining a smooth endocardium is clearly an easier step if compared to the reconstruction of a detailed geometry which includes papillary muscles and trabeculae carneae.
2. use the *simple‐array* algorithm with a threshold *σ* = 1.5 *mm* to tag all the papillary muscles and trabeculae carneae;
3. use the *drawing* algorithm to depict interactively an additional tag on the base of the left‐ventricle. Note that this location is just an example. Such interactive tag can be useful when high resolution is needed on a part of the mesh, for instance to study a particular pathological region.

The two additional tags created are shown in Figure 14C. We remark that both the *simple‐array* algorithm and the *drawing* algorithm generate tags characterized by an irregular boundary (see also Figure 7). However, in this case, the two additional tags are generated just to define a specific mesh‐size function on them and after this operation they can be deleted. Moreover, since no clipped or irregular triangles are generated, this choice help in making the successive remeshing faster.

At this stage, we can define the mesh‐size function *h* independently on each tag. The general idea is to define *h* as a constant value on some regions and as a combination of functions depending on relevant geometric quantities on some others. For the latter case, proceeding in the same way as in Section 2.3.3, we first compute the mean curvature (using *vmtk*) and the thickness of the muscle (using the *surface‐thickness* algorithm, Section 2.3.2). Then, the mesh‐size function is computed using the *surface‐mesh‐size* tool as follows:

1. using the *array* algorithm, initialize *h* on the whole surface as a function of the thickness *h*_*τ*_. In particular, we modify the following parameters in Equation (12): *α* = 0.5, *m* = 0.5 *mm*, *M* = 3 *mm*. This initialization will remain active only in the epicardium, while in the following steps we will modify the mesh‐size in all the other tags. We remark that, thanks to the chosen value of *α*, *h*_*τ*_ guarantees at least two elements per‐thickness;
2. using the *array‐combination* algorithm, modify *h* at the endocardium as a function of both the thickness and the curvature. Here, we choose the same parameters as in the example of Section 2.3.3 (Figure 10);
3. using the *array* algorithm, modify *h* only in the tag which identifies the papillary muscles and the trabeculae carneae. Here, we define the mesh‐size dependent only on the mean curvature. In particular, setting *α* = 0.2, *β* = −0.5, *m* = 0.2 *mm*, and *M* = 1 *mm*, we recover a smaller mesh‐size in this region with respect to the one on the endocardium.
4. using the *constant* algorithm, set the mesh‐size equal to 0.75 *mm* on the valvular annulus and equal to 0.5 *mm* on the tag drawn on the left‐ventricular base. The former value guarantees that at least two elements appear along the width of each annulus.

**FIGURE 15:**
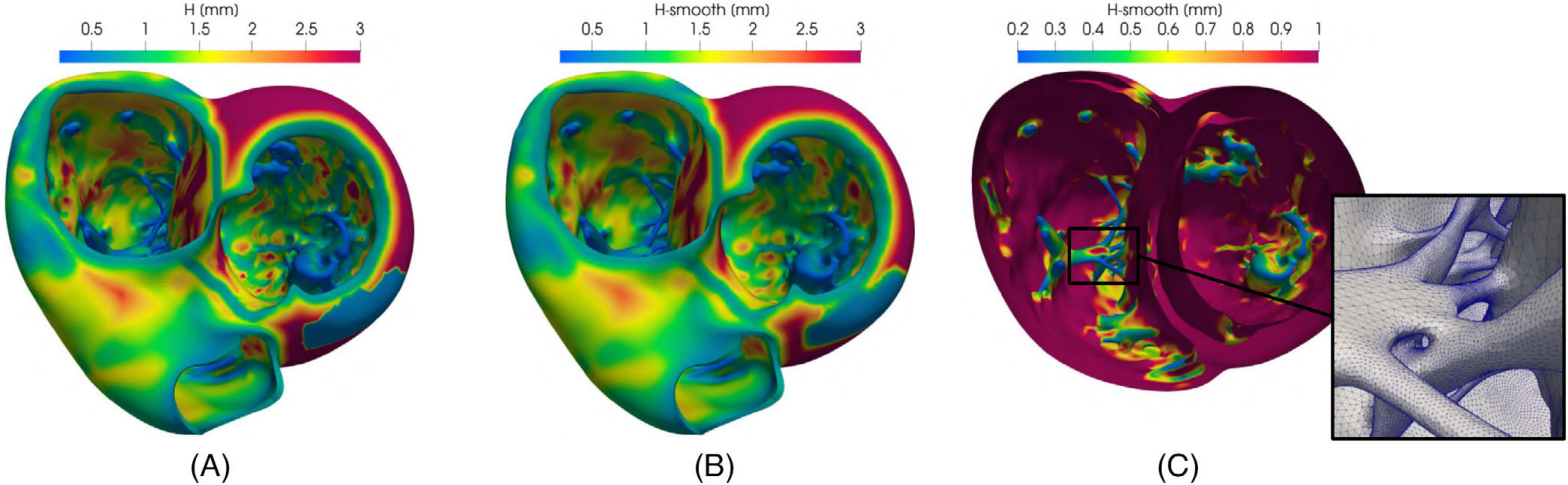
Generation of the myocardium volumetric mesh: (A) the piecewise mesh‐size *h*; (B) the smoothed version $\hat{h}$ after array smoothing operation; (C) the same $\hat{h}$ saturating the scale in order to highlight elements where the mesh‐size is lower than 1 mm and a zoom on the resulting final mesh

The tool gives as output both the final mesh‐size *h*—defined piecewise on each tag—and its smooth version $\hat{h}$, as shown in Figure 15A,B, respectively. For instance, this mesh‐size smoothing is evident by comparing *h* and $\hat{h}$ on the boundary of the tag drawn on the ventricular base. Clearly, the smoother mesh‐size is preferable in order to avoid sharp changes on the size of the final volumetric elements. As a technical note, we also remark that the modification of the initial mesh‐size can be achieved using the *surface‐mesh‐size* tool in two different ways: on the one hand, they can use a graphical interface in order to select the set of tags to be modified and to review all the performed changes; on the other hand users can perform this operation without interaction, by running the algorithm more than one time and localizing its action each time on a different subset of tags. The latter case is suggested when, once the desired parameters have been set, the user wants to automatically process a large quantity of surfaces.

In Figure 15C, we highlight the regions where the final smooth mesh‐size function $\hat{h}$ is lower than 1 *mm*. These regions are located only where they are required by the complexity of the geometry, mainly on the papillary muscles and the trabeculae carneae. Even in these parts, a mesh‐size lower than 0.5 *mm* appears only on the smallest trabeculae or on the most curved zones. Thus, remeshing the surface following this mesh‐size function and generating the volumetric mesh accordingly will produce an optimized volumetric mesh, where small elements appear only where they are really necessary. A zoom on this final mesh—made of about 300 *K* triangular surface elements and 1.1 *M* volumetric tetrahedral elements—is shown in Figure 15C.

### 3.3 A fluid‐dynamics mesh of the left heart

In this section, we provide an example of fluid‐dynamics mesh generation of the whole left‐heart. This example is mainly conceived to demonstrate the robustness of our *surface‐connection* algorithm (Section 2.1.1). Indeed, this is a crucial algorithm of our work since it is used internally in the *boolean‐connection* (Section 2.1.2), the *harmonic‐connection* (Section 2.1.3), and the *mesh‐connection* (Section 2.4.1) algorithms. As input, we use once again surfaces coming from the *Zygote* model^15^: the left‐ventricular endocardium ∑_LV_, the left‐atrial endocardium ∑_LA_, and the internal surface of the aortic root ∑_AR_. Similar input can be reconstructed from different segmentations or when each chamber is reconstructed from a specific medical image. Although we neglect the papillary muscles and the trabeculae carneae, by properly defining the mesh‐size as in Section 3.2, the described pipeline can be naturally extended to the detailed case.

**FIGURE 16:**
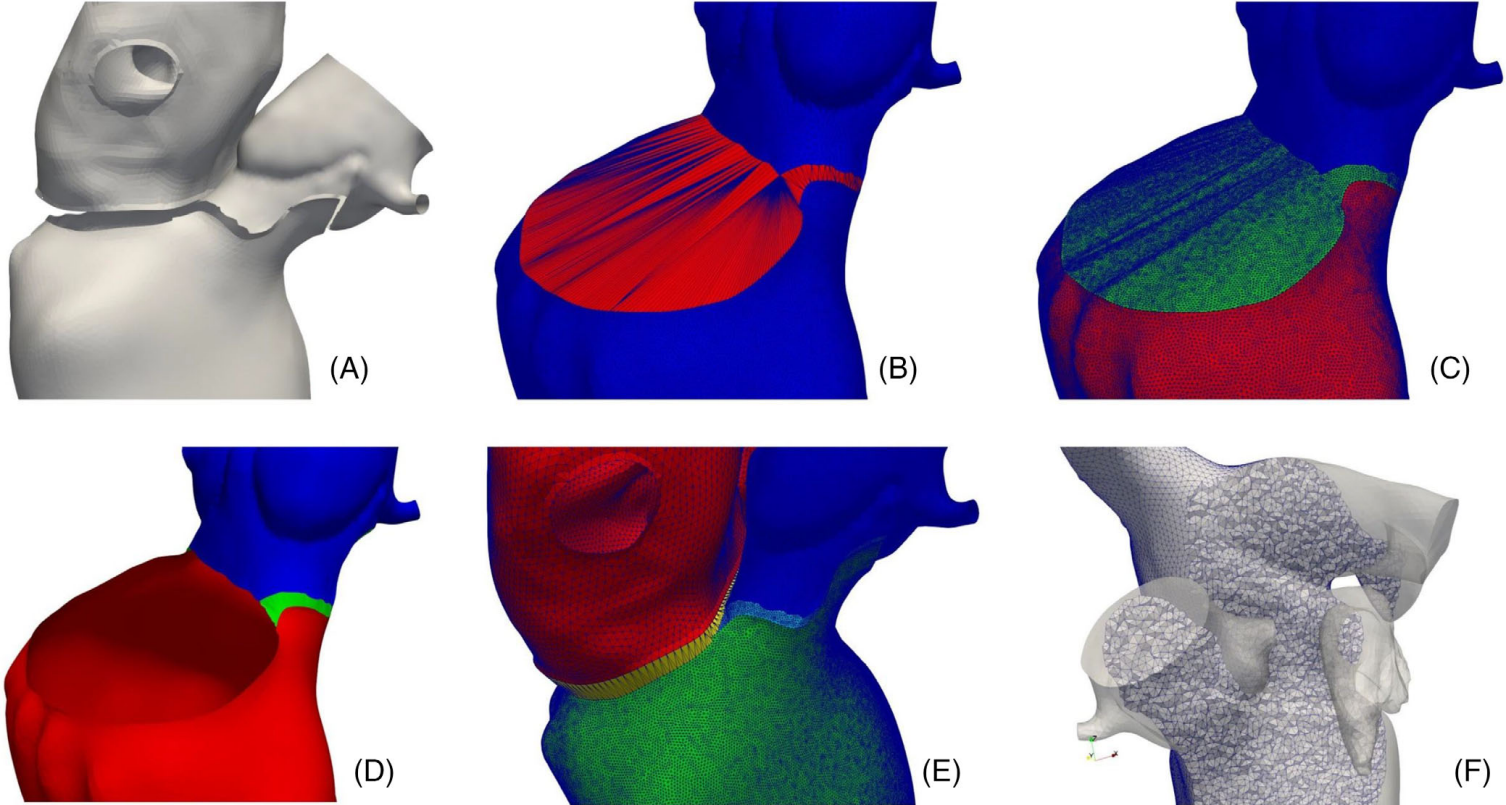
Generation of a fluid‐dynamics mesh of the left heart: (A) the starting internal surfaces of the left ventricle (LV), the left atrium (LA), and the aortic root (AR), divided by a small gap; (B) connecting the LV with the AR; (C) remesh the connection; (D) open the mitral valve ring; (E) connect the LV with the LA and remesh the connection; (F) generate the volumetric mesh

These input surfaces are divided from each‐other by a gap, as shown in Figure 16A. The difficulty of this connection is that the left ventricle is characterized by a unique hole where both the mitral valve and the aortic root are connected. Indeed, the anterior leaflet of the mitral valve is in fibrotic continuity with the aortic outflow tract. In order to connect the three organs, we proceed as follows:

1. using the *surface‐connection* algorithm, join the left‐ventricle endocardium with the aortic root. This step also caps the mitral orifice, as shown in Figure 16B;
2. remesh the just created connection recovering the regularity of the triangles and assign a specific tag to the aorta and the ventricle using the *surface‐tagger* tool, *connectivity* algorithm (Figure 16C);
3. recreate the mitral orifice, as shown in Figure 16D. This step can be done manually—using the local correction tool of *vmtk*—or automatically—clipping the surface by exploiting a signed distance from the mitral annulus;
4. using the *surface‐connection* algorithm, connect the geometry to the left atrial endocardium (Figure 16E) and remesh as desired the connection ring.

We underline that the connection performed as the first step generates abnormally stretched triangles, but at the same time it creates a cap which perfectly matches with the annulus shape. This demonstrates the robustness of the *surface‐connection* algorithm and its ability to produce the expected result also when the two rings to be connected vary their distances and are made of a very different number of points. As a final step, like in the other examples, the desired mesh‐size can be defined on the surface and, accordingly, the volumetric mesh can be generated. The example shown in Figure 16F, is made of about 400 *K* elements. However, for this smooth geometry, coarser or finer meshes can be easily generated by playing with the mesh‐size.

### 3.4 Examples of numerical simulations

**FIGURE 17:**
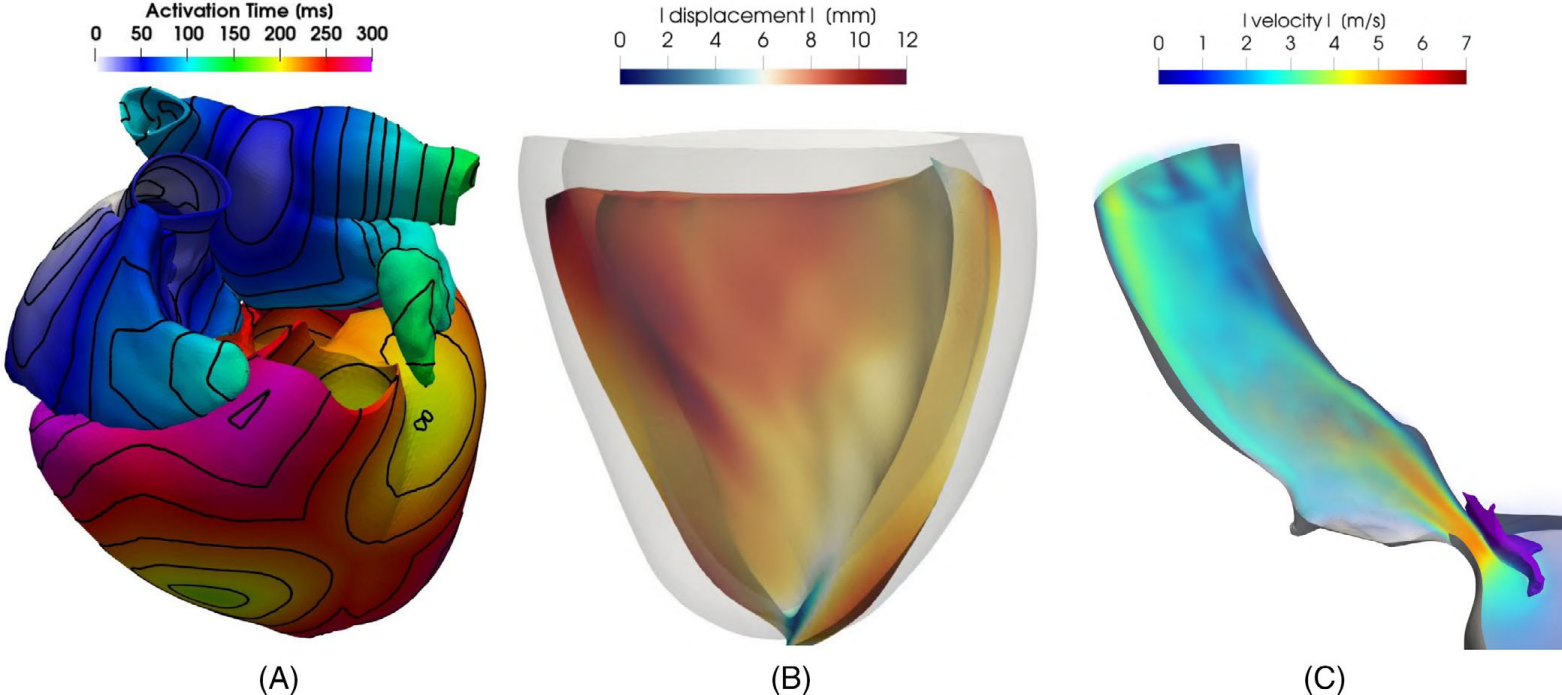
Some examples of numerical studies of the cardiac function performed on meshes generated with the tools proposed in this paper: (A) the electrical activation time of an electrophysiology simulation of the whole four‐chambers heart, see Piersanti et al^91^; (B) the resulting displacement field at the end of systole of a left‐ventricular electro‐mechanical simulation, see Regazzoni et al^92^; (C) the velocity field at the end of systole of a patient‐specific hemodynamics simulation of the systolic anterior motion of the mitral valve, see Fumagalli et al^28^

The algorithms proposed in this paper have already provided the baseline of several works concerning the numerical simulation of the cardiac function. In Figure 17 we show some examples of these studies focused on different aspects of the heart modeling:

1. Piersanti et al^91^ proposed new methods for the cardiac fibers generation. In this work electrophysiology simulations on a four‐chambers cardiac geometry were performed, as shown in Figure 17A. This complex mesh has been generated by exploiting most of the proposed algorithms of this paper, especially the ones concerning tagging, connections, and mesh‐size function definition;
2. in their multiscale study of the cardiac active mechanics, Regazzoni et al^92^ used our algorithms to generate a left‐ventricular mesh for electro‐mechanical simulations, as shown in Figure 17B;
3. Fumagalli et al^28^ performed a computational hemodynamics study of the left heart to assess the pathological systolic anterior motion of the mitral valve, as shown in Figure 17C. They used both the *harmonic‐connection* and *harmonic‐extension* algorithms in order to connect the patient‐specific ventricle with a template geometry and to extend the image‐based displacement field on the whole domain. They also took advantage of the tagging, mesh‐size and local refinement tools.

Moreover, the meshing tools proposed in this paper have also been used in other works regarding the cardiac electrophysiology,^93^, ^94^ the cardiac electromechanics,^95^ and the cardiac perfusion.^96^ Finally, we remark that also electro‐mechanics and electro‐mechano‐fluid models of the whole heart are currently under development.

The variety of these numerical studies demonstrates the flexibility of the proposed algorithms that, being connectible with each other into personalized pipelines, can be adapted to the specific needs of different numerical models of the cardiac function.

## 4 DISCUSSION AND CONCLUSIONS

In this paper, we proposed a set of new algorithms and tools for polygonal surface processing and mesh generation in the context of numerical simulations of the different physical processes that characterize the cardiac function. Our study is motivated by the complexity of the shape of the human heart and by the multi‐physics and multi‐scale cardiac processes that can be simulated numerically. This complexity reverberates in the mesh generation pipeline which, depending on the aspects of the cardiac function to be studied, can consist on a large amount of time‐consuming steps, especially in terms of manpower needed. As a consequence, a general unique pipeline for the cardiac mesh generation is very likely unsuitable, since the procedure strictly depends on the specific cardiac physics or region to be studied. For this reason we proposed various independent algorithms and tools which can be combined in different and flexible ways depending on the specific interest. This allows the creation of ad‐hoc mesh generation pipelines for each cardiac application, ranging from the simplest case of a single physics simulation in a single cardiac chamber to the most complex ones of an electro‐mechano‐fluid model of the whole heart.

We developed our algorithms as an extension of the *vascular modeling toolkit (vmtk)* library.^66^ Indeed, we use *vmtk* both for the already existing algorithms—originally designed for the vascular applications—and for its ability of nesting multiple algorithms in a single pipeline. We presented the algorithms grouping them into four different topics: polygonal surface processing, boundary tags definition, array processing and mesh‐size definition, volumetric mesh processing. We summarize hereafter the main results obtained for each of them.

1. **Polygonal surface processing**. In order to perform patient‐specific simulations of the cardiac function, a mesh generation pipeline usually starts from unprocessed surfaces reconstructed from medical images. Moreover, the various parts of the heart—for instance the endocardium, the epicardium, or each cardiac chamber—are usually reconstructed separately, producing disconnected or intersecting surfaces. In this context, we proposed three different algorithms to connect separated surfaces into a unique triangulation ready for the volumetric mesh generation:
  - the *surface‐connection* algorithm (Section 2.1.1) connects two disconnected surfaces by automatically generating a triangulation between two of their boundary rings. The robustness of the proposed technique is demonstrated in the example of mesh generation pipeline shown in Section 3.3, where we joined the endocardium of the left ventricle with the endocardium of the left atrium and the aortic root (see Figure 16);
  - the *boolean‐connection* algorithm (Section 2.1.2) performs boolean operations between intersecting polygonal surfaces by producing, differently than with standard algorithms,^80^ a regular triangulation ready for the volumetric mesh generation. An example applied to the connection of the left‐ventricular endocardium and epicardium is shown in Section 2.1.2, see also Figure 4.
  - the *harmonic‐connection* algorithm (Section 2.1.3) produces a continuous surface by deforming a boundary of an input surface into a boundary of a reference surface and by harmonically extending this deformation on the whole input surface. The conformity of the output triangulation is guaranteed by the internal usage of the *surface‐connection* algorithm. In Section 2.1.3 we showed the application of this technique to the deformation, at the valvular annulus, of a left‐atrial template geometry into a patient‐specific left‐ventricle, see Figure 5.

We also proposed an algorithm to inflate a surface representing a structure—for example, a cardiac muscle—in order to correct regions where the muscular thickness is unrealistically small (the *surface‐thickening* algorithm, Section 2.1.4), as shown for the right ventricular outflow tract in Figure 6.

2. **Boundary tags definition**. Usually, cardiac boundaries are not sharp edges easy to be localized. Thus, tagging cardiac polygonal surfaces in order to identify specific regions—for instance for the imposition of boundary conditions—is not always a trivial operation. The *surface‐tagger* tool (Section 2.2) generates tags by exploiting a function defined on it, for instance a distance from an object. In particular, the boundary of the new tag is built at the level where the function assumes a user‐defined value. The resulting tags can have different features depending on the specific algorithm used:
  - the *simple‐array* algorithm does not modify the triangulation, by limiting its action to the assignment of a tag to the already existing triangles. Thus, the boundary of the generated tag is typically irregular;
  - the *clip‐array* algorithm splits the original triangles into more elements, in order to accurately define the tag exactly at the level where the input function assumes the user‐defined value. Thus, this algorithm generates irregular triangles and must be followed by a surface remeshing to recover the regularity of the triangulation;
  - the *harmonic‐array* algorithm, instead, is a novel approach that moves the points of the triangulation thanks to a harmonic map by ensuring that some of them lie on the user‐defined level of the input function. In this way, the generated tag is accurate and the regularity of the triangulation is preserved.

A comparison of the results of the three algorithms has been graphically reported in Figure 7. Although the *harmonic‐array* algorithm shares the advantages of both the *simple‐array* and the *clip‐array* algorithms without any drawback, the other two algorithms can be used for some mesh‐generation pipelines, like in the example shown in Section 3.2 where they are used to generate on a detailed biventricular geometry the tags of valvular annulus and the papillary muscles, respectively (see Figure 14).

3. **Array processing and mesh‐size definition**. Through the *surface‐mesh‐size* tool (Section 2.3.3), we defined the mesh‐size as a function of relevant geometric quantities. Additionally, we introduced the flexibility of setting it in a different way on each tag. This allows, for instance, to generate volumetric meshes characterized by small elements only where they are required by the geometric complexity or where the user desires a particular accuracy. An example of this strategy is discussed in Section 3.2 for a detailed biventricular geometry (see Figure 15). In this context, we also proposed new algorithms to manipulate arrays defined on the surface—by the *harmonic‐extension* algorithm (Section 2.3.1)—or to compute relevant geometric quantities for the heart—by the *surface‐thickness* algorithm (Section 2.3.2).
4. **Volumetric mesh processing**. Once the mesh‐size is defined on the surface, the volumetric tetrahedral mesh can be generated using well‐known robust algorithms.^62^ However, additional processing can be required for some applications. For instance, the proposed *mesh‐refinement* algorithm (Section 2.4.2) performs an a priori local refinement of the mesh near a region of interest, as shown in Figure 16 for a hemodynamics mesh of the left‐heart refined near the mitral valve. We also proposed a possible strategy to exploit the *tet‐hex* algorithm (Section 2.4.3) in order to generate hexahedral meshes from tetrahedral ones, trying to limit the distortion of the final elements (see Figure 13 for an application to a ventricular geometry). Finally, the proposed *mesh‐connection* algorithm (Section 2.4.1) is of particular interest for the mesh generation of the whole heart. Indeed, it is able to generate a volumetric mesh between two corresponding tags on the surfaces of two volumetric meshes, as shown in Figure 11 for an idealized geometry. This can be applied, for instance, to the generation of the volumetric meshes of the valvular annulus that connect the ventricles to the atria. In addition, starting from the internal surface of the connected meshes, a conforming mesh for the fluid‐dynamics simulations can be easily generated. Moreover, the tool also assigns a specific tag to each different volume. This feature can help to assign specific physical properties to each region, as it is required by multi‐chambers electro‐mechanical models.^45^ All these considerations make this algorithm of fundamental importance for the generation of the whole heart mesh, essential for the full electro‐mechano‐fluid model of the cardiac function.

The proposed algorithms have been combined into ad‐hoc pipelines to test them in some complex cardiac applications, like the mesh generation of a fully‐detailed ventricular geometry including the papillary muscles and the trabeculae carneae (Section 3.2) or a complete fluid‐dynamics mesh of the left‐heart (Section 3.3). Moreover, as discussed in Section 3.4, they have already been successfully used in other papers to address a broad range of applications.^28^, ^91^, ^92^, ^93^, ^94^, ^95^, ^96^ These examples highlight the flexibility of these tools and their ability to be easily applicable to a large variety of cardiac mesh generations through the building of ad‐hoc application‐dependent pipelines. Despite these pipelines can be made up of many steps, once the procedure for the specific application has been established, it can be automatically applied to large datasets, potentially having a strong impact in clinical studies or in the creation of virtual cohorts of heart models.^73^

## CONFLICT OF INTEREST

The authors declare no potential conflict of interests.

## Endnotes

^*^
http://www.vmtk.org/.

^†^
https://github.com/marco-fedele/vmtk.
